# Cholesterol-mediated Lysosomal Dysfunction in *APOE4* Astrocytes Promotes α-Synuclein Pathology in Human Brain Tissue

**DOI:** 10.1101/2025.02.09.637107

**Published:** 2025-02-14

**Authors:** Louise A. Mesentier-Louro, Camille Goldman, Alain Ndayisaba, Alice Buonfiglioli, Rikki B. Rooklin, Braxton R. Schuldt, Abigail Uchitelev, Vikram Khurana, Joel W. Blanchard

**Affiliations:** 1. Icahn School of Medicine, Mount Sinai, New York, NY, USA; 2. Nash Family Department of Neuroscience, Mount Sinai, New York, NY, USA; 3. Friedman Brain Institute, Mount Sinai, New York, NY, USA; 4. Ronald M. Loeb Center for Alzheimer’s Disease, Mount Sinai, New York, NY USA; 5. Black Family Stem Cell Institute, Mount Sinai, New York, NY, USA; 6. Aligning Science Across Parkinson’s (ASAP) Collaborative Research Network, Chevy Chase, MD 20815, USA; 7. Ann Romney Center for Neurologic Diseases, Brigham and Women’s Hospital, Boston, MA, USA; 8. Division of Movement Disorders, American Parkinson Disease Association (APDA) Center for Advanced Research and MSA Center of Excellence, Department of Neurology, Brigham and Women’s Hospital, Boston, MA, USA; 9. Harvard Medical School, Boston, MA, USA; 10. The Broad Institute of MIT and Harvard, Cambridge, MA, USA; 11. Harvard Stem Cell Institute, Cambridge, MA, USA; 12. Macaulay Honors College at Hunter College, New York, NY, USA; 13. These authors contributed equally; 14. Lead contact

**Keywords:** α-Synuclein, iPSC-derived brain model, Lewy Body Dementia, Alzheimer’s Disease, Astrocytes, *APOE4*, Lysosomal dysfunction, Cholesterol metabolism, Neurodegeneration, miBrain

## Abstract

The pathological hallmark of neurodegenerative disease is the aberrant post-translational modification and aggregation of proteins leading to the formation of insoluble protein inclusions. Genetic factors like *APOE4* are known to increase the prevalence and severity of tau, amyloid, and α-Synuclein inclusions. However, the human brain is largely inaccessible during this process, limiting our mechanistic understanding. Here, we developed an iPSC-based 3D model that integrates neurons, glia, myelin, and cerebrovascular cells into a functional human brain tissue (miBrain). Like the human brain, we found pathogenic phosphorylation and aggregation of α-Synuclein is increased in the *APOE4* miBrain. Combinatorial experiments revealed that lipid-droplet formation in APOE4 astrocytes impairs the degradation of α-synuclein and leads to a pathogenic transformation that seeds neuronal inclusions of α-Synuclein. Collectively, this study establishes a robust model for investigating protein inclusions in human brain tissue and highlights the role of astrocytes and cholesterol in *APOE4*-mediated pathologies, opening therapeutic opportunities.

## Introduction.

Alzheimer’s disease (AD) is canonically associated with amyloid-β and tau pathology^1^. However, neuronal intracellular inclusions of aggregated α-synuclein (α-Syn) are present in 50–90% of AD cases^2–5^. Phosphorylated α-Syn often aggregates forming Lewy Bodies and Lewy neurites, which are frequently found in the brains of individuals with AD^2–4^. The occurrence of α-Syn inclusions with amyloid-β and tau exacerbates neurodegeneration particularly in brain regions associated with memory and executive functions^5–10^. Indeed, clinical studies show that AD patients with α-Syn pathology exhibit faster cognitive decline compared to those with only amyloid-β and tau pathology^11,12^. The strongest genetic risk factor for late-onset AD, *APOE4*, significantly increases both the prevalence and severity of α-Syn pathology in AD^12,13^ and is one of the most well replicated genetic risk factors for Lewy Body Dementia (LBD)^14,15^. However, the mechanisms by which genetic factors like *APOE4* influence the presence and severity of α-Syn pathology are largely unclear. Insight into the mechanisms underlying non-amyloid-β co-pathologies could lead to much needed therapeutic and diagnostic opportunities in AD and LBD.

The development of model systems that faithfully recapitulate α-Syn pathology and the genetic and environmental context of the human brain is essential to uncovering how ancillary genetic factors, such as APOE4, modify α-Syn-driven neurodegeneration. Addition of pre-formed fibrils (PFFs) of α-Syn are commonly employed in mice^16,17^ and *in vitro* models^18–20^ to induce α-Syn pathology. PFFs are reported to spread across the brain in a prion-like manner corrupting endogenous α-Syn and promoting the propagation of pathology^18,21,22^. However, animal models and *in vitro* systems with α-Syn PFFs are plagued with high variability and often fail to induce phosphorylated α-Syn-rich inclusions while having significant biohazards concerns from the use of prion-like particles^23^. This highlights the need for more physiological models that do not rely on PFFs and can explore multicellular mechanisms of disease. For instance, the induction of neurodegenerative phenotypes in cells cultured in traditional two-dimensional (2D) conditions may be limited by a chemical and mechanical microenvironment that is vastly different from *in vivo* conditions^24^. In contrast, three-dimensional (3D) *in vitro* systems have proven more efficient than 2D systems at replicating key pathological features such as amyloid-β plaques and tau tangles^25,26^. Combining genetic approaches with 3D tissue engineering is a promising alternative for developing more physiological models of neurodegenerative disease.

We recently developed the human **m**ulti-cellular **i**ntegrated **Brain** (**miBrain**), a fully induced pluripotent stem cell (iPSC)-derived 3D human brain tissue that incorporates neurons, glia, and microvasculature into an anatomically precise tissue capable of modeling neurodegeneration in a dish^27^. The miBrain is an *in vitro* human brain tissue containing the major cells and tissues found in human brain tissue including a blood-brain barrier, active neurons with oligodendrocytes, and the brain immune cells- such as microglia and astrocytes. The miBrain can be generated from patient-derived iPSCs. Because it is an engineered tissue, this permits the generation of genetically mixed tissue. For example, coupled with CRISPR-edited iPSC lines, we can generate genetically identical miBrains with AD risk genes such as *APOE4* in specific cell types. We have developed methods to model neuropathological phenotypes associated with amyloid-β and tau in the miBrains^27^. Here, we expanded the miBrain technology, by first developing methods to cryopreserve the miBrain, enabling the production of large batches of full or partial miBrains and dramatically reducing the batch-to-batch variability and improving scalability of the model system. Leveraging this advanced approach, we developed the first highly reproducible model of α-Syn neuropathological phenotypes in an iPSC-derived human brain tissue. We then apply the miBrain to investigate the mechanisms by which *APOE4* promotes increased α-Syn phosphorylation and aggregation in human brain tissue. Our results revealed that *APOE4* increases α-Syn phosphorylation and neuronal inclusions via non-cell-autonomous mechanisms driven by lipid accumulation in APOE4 astrocytes. Our results establish robust methods for modeling α-synuclein pathological phenotypes in human brain tissue and highlight a causal role of astrocytes and lipids in *APOE4*-mediated α-Syn pathologies, opening new therapeutic opportunities AD, LBD, and other neurodegenerative diseases.

## Results

### Induction of α-Syn intracellular inclusions in a multi-cellular integrated Brain (miBrain) tissue.

**Extended Data Figure 1.**
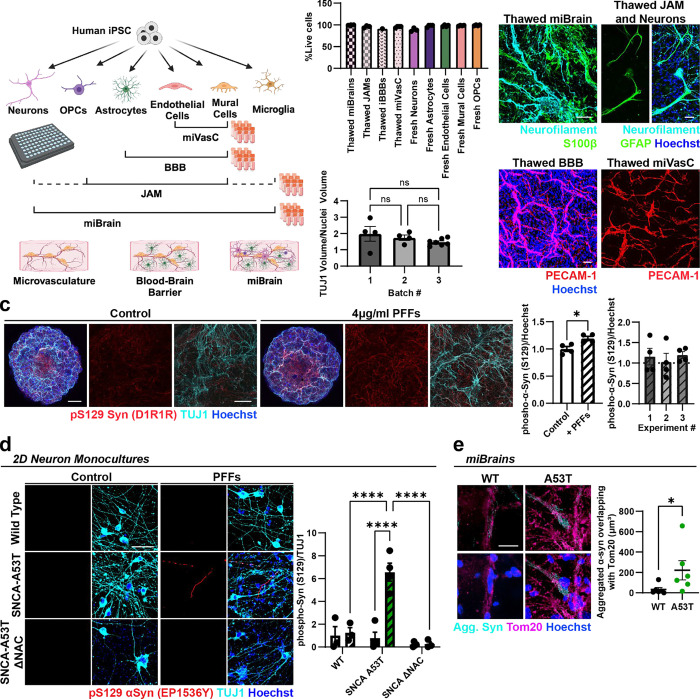
miBrain cryopreservation and development of α-synuclein intracellular inclusions. **a.** Cartoon of the cryopreservation approach to preserve miBrains or smaller tissue units (miVasC: microvascular combo; BBB: blood-brain barrier; JAM: just add missing cell type). **b.** Top: cell viability upon thawing the cryopreserved tissue compared with fresh cells harvested and counted. Bars represent cell viability (%), and error bars represent standard deviation (n = 3 biological replicates). Bottom: quantification of the ratio between neurons and nuclei volume in 18 days-old thawed miBrain tissue from three different batches. Bars represent ratio between TUJ1 and Hoechst volumes, and error bars represent standard error (n = 3 batches with 4 to 8 biological replicates each). Representative images of two-week-old, thawed tissue stained with markers of neurons (cyan; neurofilament), astrocytes (green; S100b, GFAP), and endothelial cells (red; PECAM-1). Scale bars: 50 μm. **c.** Representative images of miBrains exposed to α-synuclein PFFs for 48h and stained at two weeks. Scale bars: 500 μm and 50 μm on lower and higher magnification, respectively. The levels of α-Syn phosphorylated at Serine 129 (pS129, red) were significantly increased in miBrains exposed to PFFs. Bars represent the intensity of pS129-Syn immunostaining normalized by nuclei and by control. Left graph shows the quantification of the experiment represented by the images. Error bars represent standard error (n = 4 biological replicates). P-values were calculated using an unpaired t-test. Right graph shows the quantification for the PFF treated group in three different experiments (n = 4 biological replicates each). Error bars represent standard error. Only experiments 1 and 2 had mean levels higher than control. Point dispersion in the bar graphs display high variability. **d**. Representative images of pS129 α-Syn in neurons generated from SNCA-A53T iPSCs, wild type (WT) iPSCs, or SNCA-A53T iPSCs without the non-amyloid component (NAC) domain. SNCA-A53T neurons had increased pS129 only in the presence of PFFs. Bars represent neuron volume, and error bars represent standard error (n = 4 biological replicates). P-values were calculated by 2-way ANOVA followed by a Tukey test. Scale bars: 50 μm. **e.** In A53T miBrains, the overlap between aggregated α-synuclein and Tom20 was significantly increased. Dot plot represents the median volume of aggregated α-Syn overlapping with Tom20 (n = 4 biological replicates). P-value was calculated using a Mann-Whitney test. Scale bar: 25 μm. *P < 0.05, **P < 0.01, ***P < 0.001, ****P < 0.0001.

**Figure 1.**
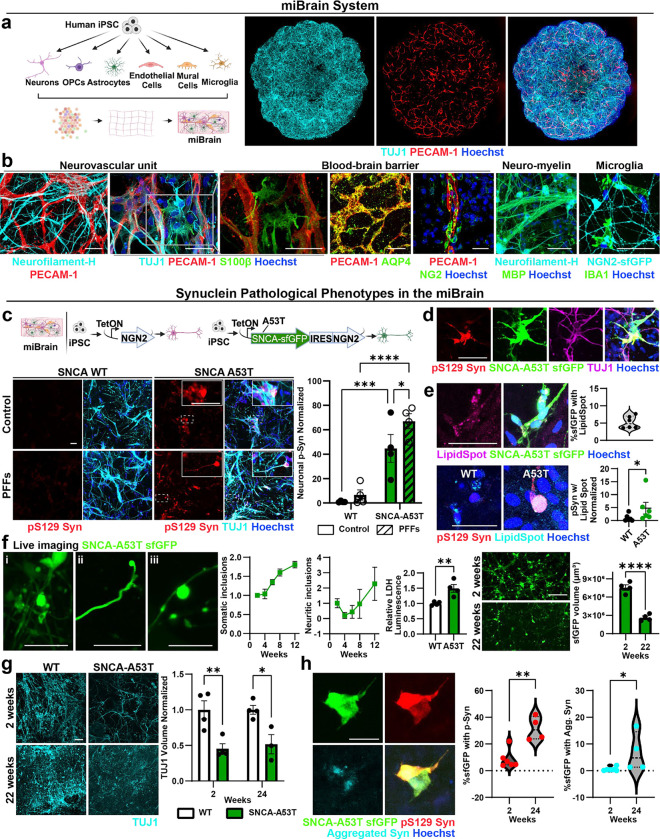
Induction of α-Syn intracellular inclusions in a multi-cellular integrated Brain (miBrain) tissue. **a**. Cartoon and representative images of our **m**ulticellular **i**ntegrated human **brain** (miBrain) tissue generated from human iPSCs differentiated into six brain cell types, including neurons, glia, and vascular cells. Four-week-old miBrains showing neurons (TUJ1, cyan) and vascular networks (PECAM-1, red). Scale bar: 500 μm. Nuclei: blue. **b**. miBrains stained for specific markers of neurons (cyan; Neurofilament-H, TUJ1, NGN2-sfGFP), astrocytes (green; S100b, AQP4), endothelial cells (red; PECAM-1), mural cells (green; NG2), myelin (green; MBP), and microglia (green; IBA1). Scale bar: 50 μm, nuclei: blue. **c.** miBrains containing neurons generated through direct iPSC reprograming via *NGN2* expression, with or without the overexpression of *SNCA-A53T*. The tissue was cultured for a total of 18 days, with or without α-synuclein PFFs added on day 4. Immunofluorescence for neuronal marker TUJ1 and for α-Syn phosphorylated at Serine 129 (pS129) shows a robust expression of pS129-Syn in A53T neurons within the miBrains, but not in WT tissue. Scale bar: 50 μm, nuclei: blue. Neuronal pS129 expression was significantly increased in miBrains with A53T neurons. Exposure to PFFs exacerbated this effect. Bars represent means of pS129-Syn^+^ volume within TUJ1^+^ neurons normalized to control WT. Error bars represent standard error (n = 4 biological replicates). P-values were calculated using a 2-way ANOVA followed by a Fisher’s LSD test. **d**. *SNCA-A53T* was fused to small folding green fluorescent protein (sfGFP). pS129-Syn^+^ inclusions co-localize with sfGFP expression in TUJ1^+^ cells. Scale bar: 50 μm, nuclei: blue. **e**. Representative images depicting the co-localization of SNCA-A53T-sfGFP (green) and neutral lipid marker Lipid Spot (magenta) in A53T miBrains, or p-Syn (red) and Lipid Spot (cyan) in wild type and A53T miBrains cultured for two weeks. Arrowhead points to a lipid droplet within a p-Syn^+^ inclusion. The violin plot shows the percentage of sfGFP-SNCA volume occupied by the overlapping signal of Lipid Spot. The sfGFP-SNCA volume overlapped at 5.2 % ± 0.7 with lipid droplet marker Lipid Spot (mean ± standard error, n = 6 biological replicates). Dot plots represent the median overlapping volume between p-Syn and Lipid Spot normalized by nuclei (n = 5–6 biological replicates). P-values were calculated by Mann-Whitney test. Scale bar: 50 μm. **f.** Live imaging of SNCA-A53T-sfGFP in cell bodies (i), neurites (ii), and varicose-like inclusions (iii). Scale bars: 25 μm (i, ii) and 15 μm (iii). Line graphs show the number of somatic and neuritic inclusions normalized by the sfGFP volume over time (n = 4 biological replicates). Compared to WT, miBrains with A53T neurons had a significant increase in the levels of lactate dehydrogenase (LDH) in the media, indicating cell death. Bars represent mean LDH luminescence normalized to WT control, and error bars represent standard error (n = 4 biological replicates). P-values were calculated using an unpaired t-test. Representative images of live tracking of SNCA-A53T-sfGFP imaging and quantification at 2 and 22 weeks after miBrain assembly revealed a reduction in sfGFP area between 2 and 22 weeks (p < 0.0001). Bars represent mean sfGFP volume. Error bars represent standard error (n = 4 biological replicates). Scale bar: 100 μm. **g**. Representative images of miBrains with A53T or WT neurons. The volume occupied by neurons (cyan; TUJ1) was significantly reduced in miBrains with A53T neurons when compared with WT at 2 and 24 weeks. Bars represent TUJ1^+^ volume and error bars represent standard error (n = 3 biological replicates). P-values were calculated by 2-way ANOVA followed by a Tukey test. Scale bar: 50 μm. **h.** Representative images of A53T miBrains stained for pS129 α-synuclein (red) and aggregated α-synuclein (cyan) 24 weeks after assembly. Bars represent the percent volume of sfGFP-SNCA occupied by either pS129 α-Syn or aggregated α-synuclein staining. P-values were calculated by Mann-Whitney test. Scale bar: 25 μm. *P < 0.05, **P < 0.01, ***P < 0.001, ****P < 0.0001

Intracellular neuronal inclusions of phosphorylated and aggregated α-Syn have been challenging to reproduce in human brain cells or tissue, typically requiring very long maturation protocols^28^ and the concomitant use of PFFs^29^. Therefore, we sought to develop a robust, high-fidelity model of α-Syn intracellular inclusions leveraging the multi-cellular integrated Brain (miBrain). The miBrain is a human brain tissue generated by encapsulation of iPSC-derived neurons^29,30^, astrocytes^31,32^, endothelial cells^33–36^, mural cells^33,37^, oligodendrocyte precursor cells (OPCs)^38^, and microglia^39^. miBrains displayed homogenous TUJ1^+^ neuronal networks and PECAM1^+^ vascular structures (Fig. 1a). Staining for multiple markers revealed interactions and co-localization between S100β^+^ and AQP4^+^ astroglia and PECAM1^+^ vascular structures, vascular coverage with NG2^+^ mural cells, myelin basic protein (MBP)^+^/neurofilament^+^ neuro-myelin structures, and the presence of IBA1^+^ microglia in a grid-like pattern (Fig. 1b), consistent with the specific cell types encapsulated in the miBrain. In other platforms such as brain organoids, significant variability in cell composition have been reported, leading to a high-degree of experimental variability and consistency^40^. Therefore, to minimize miBrain batch-to-batch variability and increase the scalability of the miBrain, we developed methods to cryopreserve large batches of miBrain tissue containing multiple cell types at a defined ratio. Off-the-shelf cryopreserved miBrains retain more than 90% cell viability upon thaw and expressed specific cell markers two weeks after thawing into the 3D culture system (Extended Data Fig. 1a, b). The ratio of neurons to nuclei did not significantly differ between three different batches of thawed miBrain tissue (p = 0.29, Extended Data Fig. 1b).

Phosphorylation of α-Syn on S129 is the predominant pathological modification associated with α-Syn aggregation and neuronal inclusions, and, therefore, is an established method for detecting pathogenic transformation of α-Syn^41,42^. Consequently, we first investigated whether the conventionally employed α-synuclein PFFs^16–20^ can increase phosphorylated α-Syn (p-Syn) in the miBrains. Inoculating the miBrain culture media with α-Syn PFFs significantly increased p-Syn levels when compared to control miBrains (p = 0.0175, Extended Data Fig. 1c), indicating the suitability of the tissue to develop α-Syn pathological phenotypes. However, using this approach, p-Syn was mostly seen as dispersed puncta (Extended Data Fig. 1c) rather than within the typical neuronal inclusions that are hallmarks of synucleinopathies^43^. In addition, there was a high degree of variability in the presence and abundance of p-Syn (Extended Data Fig. 1c) consistent with other reports comparing the pattern of α-Syn aggregate spreading in PFF models across different research groups^17^. Therefore, we sought to identify more robust methods for inducing pathogenic α-Syn phenotypes in the miBrain.

A recent study found that iPSC-derived neurons express low levels of the gene encoding α-Syn (*SNCA)* compared to adult human brain tissue and, therefore, used *SNCA* overexpression to increase α-Syn to physiological levels and induce its phosphorylation and neuronal inclusions^29^. Thus, to achieve brain-like levels of *SNCA* and induce pathological phenotypes, we generated neurons from iPSCs with inducible expression of *SNCA* bearing the A53>T mutation (A53T), known to increase α-Syn’s aggregation propensity^29,44,45^. SNCA-A53T was fused to a small folding green fluorescent protein (sfGFP, diagram in Fig. 1c), enabling live imaging and direct visualization of α-Syn accumulation^46^. We confirmed that α-Syn phosphorylation is dependent on the α-Syn protein non-amyloid component (NAC) domain and not influenced by sfGFP (Extended Data Fig. 1d). In 2D monocultures, wild-type (WT) and A53T neurons have similar levels of p-Syn, which significantly increased in A53T neurons with the addition of PFFs (Extended Data Fig. 1d). In contrast, in the absence of exogenously added PFFs, miBrains with A53T neurons already showed a significant increase in neuronal p-Syn compared to isogenic miBrains harboring WT neurons (p = 0.0007), suggesting that the more physiological 3D environment of the miBrain is more permissive to α-Syn pathology. We found the addition of PFFs in A53T miBrains further increased p-Syn levels compared to WT miBrains with PFFs (p < 0.0001) and A53T miBrains without PFFs (p = 0.038, Fig. 1c). The SNCA-A53T-sfGFP signal co-localized with p-Syn (Fig. 1d and Supplementary Video 1; Control: 13.54% ± 2.37, PFFs: 17.79% ± 2.61, mean of the percentage of colocalization ± SEM, n = 4), indicating that SNCA-A53T undergoes phosphorylation at a similar extent with or without PFFs (p = 0.27, unpaired t-test). Although most of the p-Syn co-localized with sfGFP (Control: 86.7% ± 1.9, PFFs: 88.0% ± 1.4, n = 4), we also found p-Syn that did not co-localize with sfGFP (Control: 13.3% ± 1.9, PFFs: 12.0% ± 1.4, n = 4), indicating that endogenous, non-A53T α-Syn is also phosphorylated. This data shows that A53T miBrains developed p-Syn-rich inclusions via corruption of both induced (A53T) and endogenous (WT) α-Syn without requiring the use of PFFs.

In the human brain, lipid droplets^47^ and mitochondria^48^ are frequently observed within α-Syn^+^ inclusions. In A53T miBrains, the neutral lipid marker Lipid Spot overlapped with SNCA-A53T-sfGFP and with p-Syn (Fig. 1e). The co-localized volume between Lipid Spot and p-Syn was significantly increased (p = 0.02) in A53T miBrains compared with WT (Fig. 1e), consistent with previous reports on p-Syn^+^/Lipid Spot^+^ neurotoxic inclusions in iPSC-derived neurons^29^. Furthermore, the volume of aggregated α-Syn overlapping with mitochondrial marker Tom20 was significantly increased in A53T miBrains compared to WT miBrains (p = 0.04, Extended Data Fig. 1e), consistent with reports of aggregated α-Syn bound to mitochondria in α-Syn^+^ inclusions in the human brain^49^.

Neuronal inclusions of α-Syn present as spherical dense Lewy Bodies, less dense “pale bodies”, and Lewy neurites that can be thread-like or attain a varicose appearance^43^. Through serial live imaging, we observed somatic and neuritic presentations of the SNCA-A53T-sfGFP signal in miBrains, resembling various morphological presentations found in the human brain (Fig. 1f, i–iii). During the first 12 weeks, there was a significant increase in the number of α-Syn somatic inclusions (2 weeks: 4.7 × 10^−5^ ± 1.9 × 10^−6^, 12 weeks: 8.5 × 10^−5^ ± 4.0 × 10^−6^, number of sfGFP^+^ inclusions normalized by total sfGFP volume, p < 0.0001, unpaired t-test), and no significant changes in the number of α-Syn neuritic inclusions (2 weeks: 1.5 × 10^−7^ ± 4.4 × 10^−8^, 12 weeks: 3.4 × 10^−7^ ± 1.6 × 10^−7^, p= 0.3; Fig. 1f). *In vivo* aggregation of α-Syn within neuronal inclusions promotes neuronal death^50,51^. Soluble biomarkers of cell death (lactate dehydrogenase (LDH)) were significantly higher in media of A53T miBrains compared to WT miBrains (p = 0.0018) and the sfGFP volume was significantly reduced between 2 and 22 weeks of miBrain assembly (p < 0.0001, Fig. 1f). Consistent with α-Syn-induced neuronal death we observed a significant reduction in the volume of TUJ1 staining at 2 weeks (p = 0.002) and 24 weeks (p = 0.011) in A53T miBrains when compared to WT (Fig. 1g). By 24 weeks, A53T miBrains had significantly increased percentages of the sfGFP volume overlapping with pS129 Syn (p = 0.0095), and aggregated α-Syn (p = 0.0381) in comparison with 2 weeks miBrains (Fig. 1h), consistent with aggregation of α-Syn over time. Collectively, these results show that the miBrain can effectively model critical aspects of α-Syn pathology.

### *APOE4* increases the phosphorylation and aggregation of α-Syn in the miBrain

**Extended Data Figure 2.**
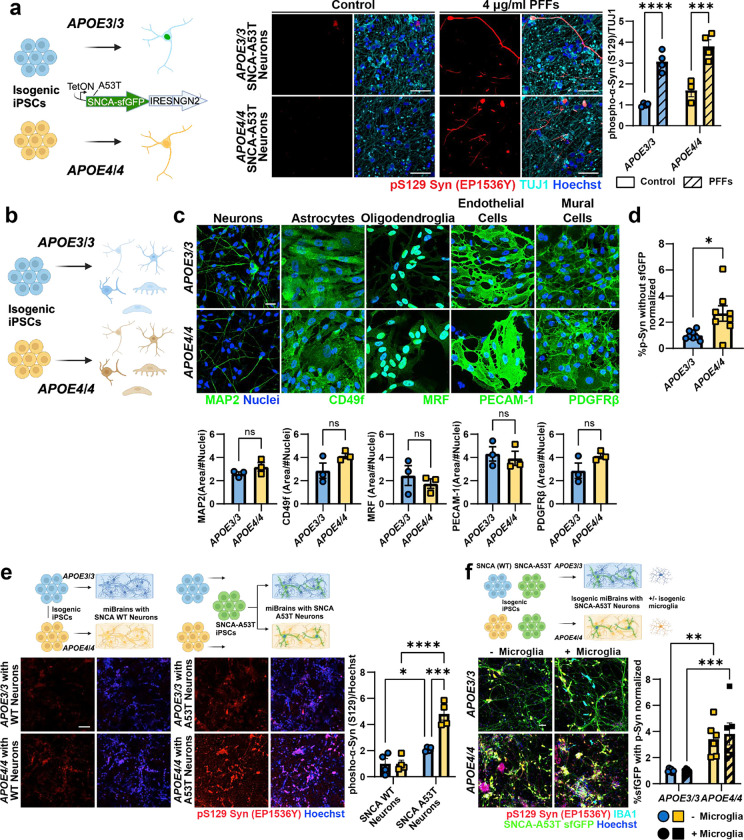
Non-neuronal cells promote α-synuclein pathological phenotypes in APOE4 tissue. **a.** Representative images of 2D *APOE3/3* and *APOE4/4* isogenic neuronal cultures overexpressing SNCA-A53T-sfGFP and exposed to PFFs for 2 weeks. Scale bar: 50 μm. pS129 was increased in both *APOE3/3* and *APOE4/4* SNCA-A53T neurons after exposure to PFFs when compared to WT controls. Bars represent mean volume of phosphorylated α-Syn per β-III tubulin volume and normalized to control *APOE3/3* neurons; error bars represent standard error (n = 4 biological replicates). **b.** Cartoon depicting the experimental paradigm for generating isogenic *APOE3/3* and *APOE4/4* cells for miBrains. **c.** Cells were harvested on the day of miBrain assembly, and a subset of the cells was plated in monocultures and fixed after 24h. Representative images of *APOE3/3* and *APOE4/4* iPSC-derived cells stained for specific markers (green) of each cell type. Nuclei: blue. Scale bar: 20 μm. Bars represent mean values of the area immunoreactive for each cell marker normalized by nuclei and error bars represent standard error (n = 3 biological replicates). P-values were calculated using unpaired t-test. **d.** *APOE4/4* miBrains had significantly higher levels of phosphorylated α-Syn outside the sfGFP volume compared with *APOE3/3*. Bars represent mean values of percent of sfGFP volume outside the sfGFP volume, normalized by *APOE3/3*. Error bars represent standard error (n = 8 biological replicates). P-values were calculated using unpaired t-test. **e.** Representative images of *APOE3/3* and *APOE4/4* miBrains with WT or SNCA-A53T-sfGFP neurons. WT miBrains were generated with isogenic *APOE3/3* or *APOE4/4* cells. A53T miBrains were generated with A53T neurons that were differentiated from an iPSC line from an *APOE3/3* donor with familial Parkinson’s disease. All other cells were isogenic between each other except for the APOE locus. Scale bar: 50 μm. The presence of the SNCA-A53T neurons significantly increased the levels of pS129-Syn, but this effect was exacerbated when the non-neuronal cells harbored *APOE4/4*. Bars represent mean volume immunoreactive for phosphorylated α-Syn per nuclei volume and normalized to *APOE3/3* miBrains with WT neurons. Error bars represent standard error (n = 4 biological replicates). P- values were calculated using 2-way ANOVA followed by a Fisher’s LSD test. **f.** Representative images of *APOE3/3* and *APOE4/4* miBrains with isogenic microglia and SNCA-A53T-sfGFP neurons. Scale bar: 50 μm. The presence of microglia in either APOE3/3 or APOE4/4 tissue did not significantly change the levels of pS129-Syn. Bars represent mean values of percent of sfGFP volume immunoreactive for phosphorylated α-Syn, and error bars represent standard error (n = 6 biological replicates per combination). P-values were calculated using 2-way ANOVA followed by a Fisher’s LSD test. *P < 0.05, **P < 0.01, ***P < 0.001, ****P < 0.0001.

**Figure 2.**
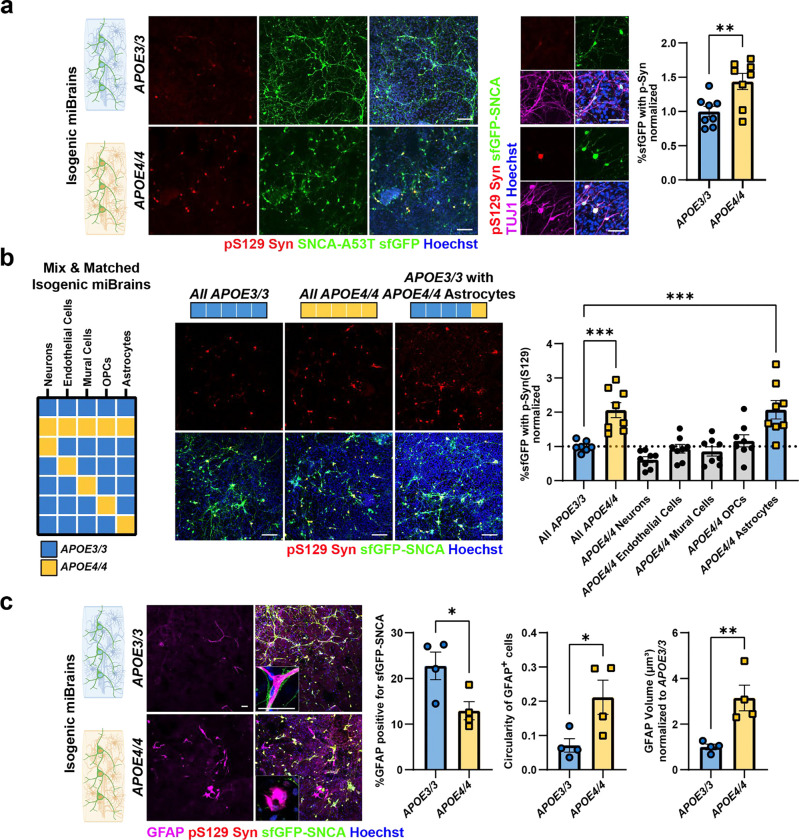
*APOE4* increases the phosphorylation and aggregation of α-Syn via astrocytes. **a.** Using CRISPR-Cas9 on an *APOE3/3* iPSC line, we generated isogenic iPSCs harboring *APOE4/4* and differentiated them into neurons with SNCA-A53T overexpression. We generated miBrains with *APOE3/3* or *APOE4/4* isogenic cells. Representative images of phosphorylated α-Syn immunoreactivity in *APOE3/3* and *APOE4/4* isogenic miBrains. Higher magnification panel depicts co-localized immunoreactivity between pS129 (red), SNCA-A53T-sfGFP (green) and TUJ1 (magenta). Scale bars: 50 μm. *APOE4/4* miBrains had significantly higher levels of pS129 (red) compared with *APOE3/3*. Bars represent mean values of percent of sfGFP volume immunoreactive for phosphorylated α-Syn, normalized by *APOE3/3*. Error bars represent standard error (n = 8 biological replicates). P-values were calculated using unpaired t-test. **b.** We generated miBrains with *APOE3/3* or *APOE4/4* isogenic cells, including miBrains with one cell type at a time harboring *APOE4/4* (yellow boxes) and the remaining cells *APOE3/3* (blue boxes). Representative images of phosphorylated α-Syn in a combinatorial screen of *APOE3/3* and *APOE4/4* isogenic cell types. *APOE4/4* and *APOE3/3* miBrains with *APOE4/4* astrocytes had equivalent levels of pS129 α-Syn (red). These levels were significantly higher than in miBrains where all cells harbored *APOE3/3* or where any cell type except astrocytes harbored *APOE4/4*. Bars represent mean values of percent of sfGFP volume immunoreactive for phosphorylated α-Syn, and error bars represent standard error (n = 8 biological replicates per combination). P-values were calculated using one-way ANOVA followed by a Dunnett’s Multiple Comparisons Test. Scale bar: 50 μm. **c.** Representative images of GFAP immunoreactivity in *APOE3/3* and *APOE4/4* miBrains. Scale bar: 50 μm. *APOE4/4* miBrain astrocytes (GFAP^+^) had reduced overlap with sfGFP-SNCA, increased circularity and significantly higher levels of GFAP (magenta) compared with *APOE3/3*. Bars represent mean values of GFAP volume and circularity and error bars represent standard error (n = 4 biological replicates). P-values were calculated using unpaired t-test. *P < 0.05, **P < 0.01, ***P < 0.001, ****P < 0.0001

*APOE4* is a genetic risk factor for α-Syn in LBD^14^ and AD^52,53^, and it is associated with increased disease severity in human studies, animal models^54,55^, and *in vitro* systems^56^. To model and investigate the mechanisms by which *APOE4* promotes α-Syn pathology, we utilized isogenic iPSC lines obtained from an *APOE3/3* individual CRISPR-edited to *APOE4/4*^57,58^. Monocultures of *APOE4/4* A53T neurons showed similar levels of p-Syn as isogenic *APOE3/3* SNCA-A53T neurons with or without PFF exposure (Extended Data Fig. 2a). Therefore, we differentiated the isogenic iPSCs into astrocytes, OPCs, endothelial cells, and mural cells observing similar cell type-specific marker expression between the *APOE3/3* and *APOE4/4* genotypes for each cell type (Extended Data Fig. 2b–c). We next generated isogenic *APOE3/3* and *APOE4/4* miBrains containing SNCA-A53T neurons. Consistent with clinical studies, *APOE4/4* miBrains with SNCA-A53T neurons showed significantly increased neuronal p-Syn compared to isogenic control *APOE3/3* miBrains (p = 0.0074, Fig. 2a). The volume of p-Syn outside the sfGFP mask was also significantly increased in *APOE4/4* miBrains compared to *APOE3/3* (p = 0.0156, Extended Data Fig. 2d), indicating that endogenous non-A53T α-Syn is also phosphorylated in the *APOE4/4* miBrains. To assess whether increased phosphorylation of α-Syn in the *APOE4/4* miBrain is a direct cell-autonomous effect of *APOE4/4* neurons, we generated miBrains that were all *APOE4/4* except for *APOE3/3* SNCA-A53T neurons. Strikingly, we observed a significant increase in p-Syn even when *APOE3/3* SNCA-A53T neurons were placed in an otherwise *APOE4/4* miBrain (p = 0.0001, Extended Data Fig. 2e).

### APOE4/4 astrocytes are responsible for increased α-Syn pathological phenotypes in human brain tissue.

Our results suggest that non-neuronal cell types are responsible for increased p-Syn in the *APOE4/4* miBrains. To identify which *APOE4* cell types promote α-Syn pathology, we generated permutations of the *APOE3/3* miBrains where each cell type was replaced with isogenic *APOE4/4* cells (Fig 2b). Consistent with previous results, all *APOE4/4* miBrain had significantly increased p-Syn staining compared to isogenic *APOE3/3* miBrains. Replacing *APOE3/3* neurons, endothelial cells, mural cells, or OPCs with their *APOE4/4* isogenic counterpart did not significantly increase p-Syn immunoreactivity in the miBrain (p = 0.99, Fig. 2b). However, selectively replacing *APOE3/3* astrocytes with isogenic *APOE4/4* astrocytes led to a significant increase in p-Syn immunoreactivity (p = 0.014) reaching levels similar to the all *APOE4/4* miBrains (Fig. 2b), suggesting that *APOE4/4* astrocytes are responsible for increasing α-Syn pathology in *APOE4* miBrain. Since microglia are implicated in the clearance of α-Syn^59,60^, we investigated the effect of the presence or absence of microglia in the isogenic miBrains. The presence of microglia in the *APOE3/3* and *APOE4/4* miBrains did not significantly alter α-Syn phosphorylation (Extended Data Fig. 2f). Astrocytes are responsible for uptake and degradation of α-Syn released by neurons^61–63^. We found that GFAP^+^ *APOE4/4* astrocytes in the *APOE4/4* miBrain have a significantly reduced overlap with SNCA-A53T-sfGFP (p = 0.0348) and adopt an amoeboid-like morphology with increased circularity (p = 0.0398) and GFAP expression (p = 0.0100) compared with *APOE3/3* miBrain astrocytes (Fig. 2c and Extended Data Videos 2 and 3), suggesting changes in astrocytic uptake of α-Syn associated with increased reactivity. Collectively, these results highlight a critical role for *APOE4/4* astrocytes in the phosphorylation and aggregation of neuronal α-Syn.

### APOE4 astrocytes have impaired processing of exogenous α-Syn.

**Extended Data Figure 3.**
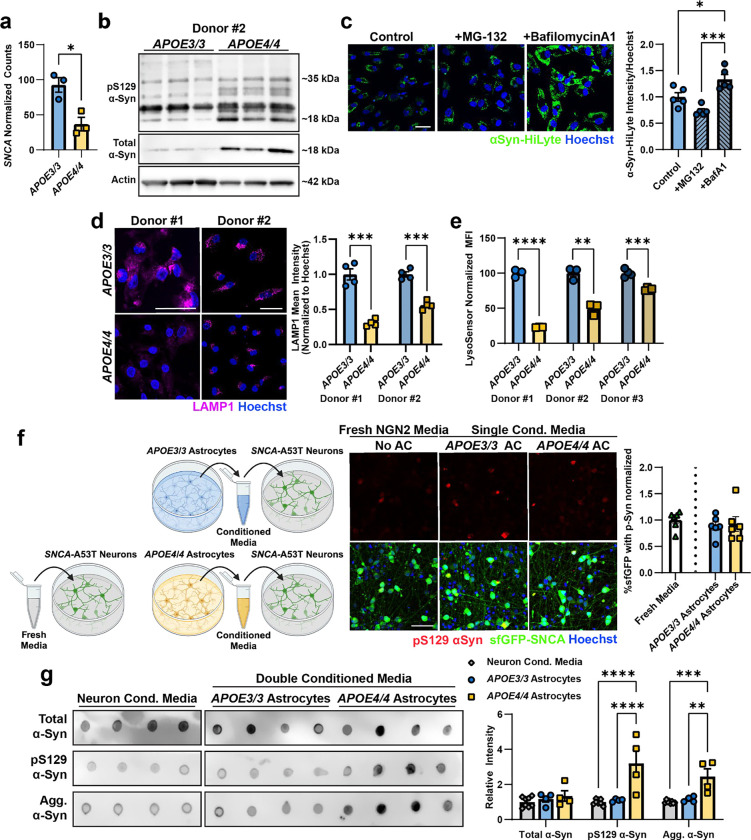
Characterization of lysosomal function and α-Synuclein uptake and accumulation in astrocytes. **a.** RNAseq analysis of isogenic, iPSC derived astrocytes for SNCA expression. Bars represent mean normalized counts and error bars represent standard error (n = 3 replicates). P-value was calculated by unpaired t-test. **b.** Western blots of *APOE3/3* and *APOE4/4* astrocytes for total α-Syn protein and phosphorylated α-Syn protein. Total and phosphorylated αSyn is increased in *APOE4/4* astrocytes. The expected band size of α-Syn monomers is approximately 18kDa. β-Actin was used as a loading control. (n = 3 replicates). **c.** Representative images of astrocytes with α-Syn-HiLyte (green) after 24 hours of uptake and 24 hours of degradation after treatment with BafilomycinA1 and MG-132. αSyn degradation is only affected by lysosomal disruption in BafilomycinA1. Bars represent the mean α-Syn-HiLyte intensity per nuclei area, normalized to control astrocytes. Error bars represent standard error (n = 5 replicates). P-values were calculated by one-way ANOVA followed by Tukey test. **d.** Representative images of LAMP1 immunostaining (magenta) in two different isogenic iPSC lines. APOE4/4 astrocytes have significantly less LAMP1^+^ lysosomes. Bars represent mean intensity per nuclei area normalized to *APOE3/3*. Error bars represent standard error (n = 4 replicates). P-values were calculated using unpaired t-tests. **e.** LysoSensor mean fluorescence intensity after 1 minute incubation measured by flow cytometry in DAPI negative cells in three different isogenic iPSC lines. *APOE4/4* astrocytes have significantly reduced LysoSensor signal compared to *APOE3/3* astrocytes. Bars represent mean values normalized to APOE3/3 and error bars represent standard error (n = 3 replicates). P-values were calculated using unpaired t-tests. **f.** Top: Schematic of the experimental paradigm generating astrocyte conditioned media from *APOE3/3* and *APOE4/4* astrocytes. Bottom: Representative images of *SNCA*-A53T neurons treated with conditioned media from naïve *APOE3/3* or *APOE4/4* astrocytes, or with fresh neuron media. There was no differential effect between conditions. Bars represent mean pS129 volume normalized by sfGFP volume. Error bars represent standard error (n = 6 replicates). P-values were calculated using 1-way ANOVA followed by a Tukey test. **g**. Dot blots of *SNCA*-A53T conditioned media or double conditioned media from SNCA-A53T neurons and isogenic *APOE3/3* or *APOE4/4* astrocytes for total αSyn, phosphorylated αSyn, and aggregated αSyn. All dots were on one blot; the image was cropped to fit for the figure. Bars represent mean intensity and error bars represent standard error (n = 4 replicates). P-values were calculated by unpaired, t-tests. All scale bars = 50 μm. *P < 0.05, **P < 0.01, ***P < 0.001, ****P < 0.0001.

**Figure 3.**
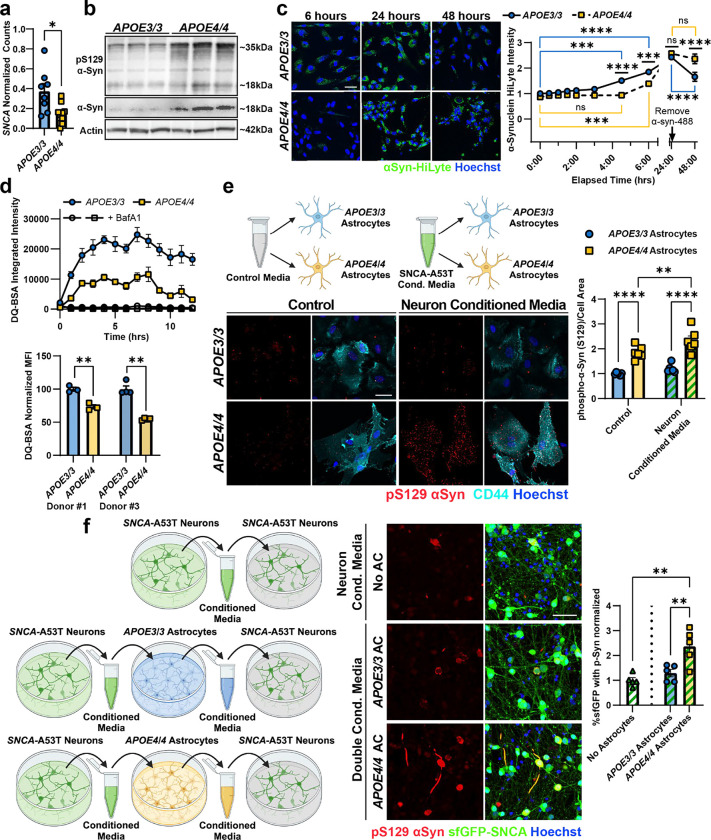
Impaired lysosomal function of *APOE4/4* astrocytes seeds α-Syn phosphorylation in neurons. **a.** Pseudobulk analysis of human astrocytes (Haney et al. 2024) for *SNCA* expression. Bars represent mean normalized psuedobulk gene count and error bars represent standard error (n = 8 APOE3/3 and n = 10 APOE4/4). P-value was calculated using unpaired t-test. **b** Western blots of *APOE3/3* and *APOE4/4* astrocytes for total α-Syn protein and phosphorylated α-Syn protein. Total and phosphorylated αSyn is increased in *APOE4/4* astrocytes. The expected band size of α-Syn monomers is approximately 18kDa. β-Actin was used as a loading control. (n = 3 replicates). **c.** Representative images of uptake and degradation of exogenous, fluorescently labeled α-Syn (green). α-Syn was removed from the culture media after 24 hours. *APOE3/3* astrocytes uptake and degrade αSyn more readily than APOE4/4 astrocytes. Data points represent mean values of α-Syn-HiLyte mean intensity normalized to nuclei area and *APOE3/3* at the first time point. Error bars represent standard error (n = 4 replicates). P-values were calculated using 2-way ANOVA followed by a Tukey test. **d.** Left: DQ-BSA Red integrated intensity per cell confluency measured over 24 hours on an Incucyte (Sartorius). BafilomycinA1 at 100nM was used as a control for non-lysosomal proteolysis of DQ-BSA. Data points represent mean values and error bars represent standard error (n = 4 replicates). Right: DQ-BSA Green mean fluorescence intensity measured in DAPI negative cell population by flow cytometry after 24 hours in two different isogenic iPSC lines. Bars represent mean values normalized to APOE3/3 and error bars represent standard error (n = 3 replicates). P-values were calculated by unpaired t-tests. **e.** Representative images of pS129 α-Syn (red) in *APOE3/3* and *APOE4/4* astrocytes after exposure to fresh or conditioned neuron media. *APOE4/4* astrocytes have more pS129 a-Syn, which is further increased upon incubation with neuronal media. Data points represent mean values of pS129 a-Syn normalized to cell area (CD44; cyan), and error bars represent standard error (n = 6 replicates). P-values were calculated using 2-way ANOVA followed by a Fisher’s LSD test. **f.** Left: schematic of the experimental paradigm generating “double conditioned media” from *APOE/3* or *APOE4/4* astrocytes previously exposed to neuronal media. Center, right: Representative images of *SNCA*-A53T neurons treated with neuron conditioned media or neuron and astrocyte conditioned media. Neurons treated with *APOE4/4* double conditioned media showed significant increase in pS129 α-Syn compared to all other conditions. Bars represent mean pS129 volume normalized by sfGFP volume. Error bars represent standard error (n = 5 replicates). P-values were calculated using 1-way ANOVA followed by a Tukey test. All scale bars = 50 μm. *P < 0.05, **P < 0.01, ***P < 0.001, ****P < 0.0001.

To investigate the mechanisms by which *APOE4* astrocytes increase the phosphorylation and aggregation of neuronal α-Syn, we first examined whether altered expression of *SNCA* in astrocytes contributes to increased abundance and phosphorylation of α-Syn in the *APOE4/4* miBrain and human brain tissue. Analysis of single-nucleus post-mortem transcriptomics data^64^ revealed that *SNCA* mRNA expression is significantly (p = 0.019) down-regulated in astrocytes from *APOE4* carriers (n = 10) compared to age-matched *APOE3/3* individuals (n = 8) (Fig. 3a). We observed a similar decrease in *SNCA* mRNA expression in *APOE3/3* and *APOE4/4* iPSC-derived astrocytes^57^ (p = 0.017) (Extended Data Fig. 3a) suggesting that transcriptional upregulation of *SNCA* in astrocytes is likely not responsible for increased phosphorylation and aggregation of α-Syn in the *APOE4* human brain tissue. However, immunoblotting astrocyte monocultures for α-Syn revealed that *APOE4/4* astrocytes have more total and phosphorylated α-Syn protein compared to isogenic *APOE3/3* astrocytes from two different individuals (Fig. 3b; Extended Data Fig. 3b) suggesting that post-translational mechanisms underlie increased α-Syn abundance in *APOE4/4* astrocytes.

Astrocytes have a well-described protective and homeostatic function of taking up and degrading neuronal α-Syn^61–63^. Given this role and our findings that *APOE4/4* astrocytes increase neuronal α-Syn phosphorylation and co-localize less with SNCA-A53T-sfGFP in the miBrain (Fig. 2c), we hypothesized that *APOE4/4* astrocytes have impaired processing of exogenous α-Syn. To investigate this, we incubated isogenic *APOE3/3* and *APOE4/4* astrocytes with fluorescently labeled α-Syn monomers (αSyn-HiLyte) for 24 hours. *APOE3/3* astrocytes showed a robust ability to take up αSyn within 4.5 hours (p = 0.001) and 6 hours (p < 0.0001) (Figure 3c). In contrast, α-Syn monomer uptake by *APOE4/4* astrocytes was significantly impaired. At 4.5 hours *APOE4/4* astrocytes had no significant (p > 0.99) intracellular α-Syn-HiLyte signal, but it began to increase by 6 hours (p = 0.005) (Fig. 3c). Despite delayed kinetics, at 24 hours *APOE4/4* astrocytes reached similar uptake levels as *APOE3/3* astrocytes. We then removed the media containing α-Syn and assessed the degradation of intracellular α-Syn-HiLyte over the following 24h. *APOE3/3* astrocytes had a significant decrease (p < 0.0001) in the α-Syn-HiLyte signal. In contrast, the signal in *APOE4/4* did not significantly decrease (p = 0.98) (Fig. 3c) suggesting that *APOE4/4* astrocytes have impaired degradation of α-Syn.

### APOE4 astrocytes have impaired lysosomal function and release pathogenic α-Syn.

Since the two main systems for the degradation of intracellular proteins are the lysosomal and proteasomal pathways^65^, we asked whether α-Syn was cleared via lysosomal or proteasomal degradation in astrocytes. We treated astrocytes with bafilomycinA1, a lysosomal inhibitor, or with MG-132, a proteasomal inhibitor. Lysosomal inhibition blocked the degradation of α-Syn-HiLyte (p = 0.02), unlike proteasomal inhibition, which had no significant effect (p = 0.07) on the degradation of α-Syn-HiLyte signal (Extended Data Fig. 3c). This confirms previous reports that α-Syn is primarily degraded through the endolysosomal pathway in astrocytes and demonstrates that *APOE4*-driven lysosomal dysfunction leads to α-Syn accumulation.

We hypothesized that the endolysosomal pathway of *APOE4/4* astrocytes is impaired in comparison to *APOE3/3* astrocytes. To assess lysosomal function, we measured the lysosome-specific proteolytic cleavage of DQ-BSA and found *APOE4/4* astrocytes have significantly decreased lysosomal proteolytic activity compared to isogenic control *APOE3/3* astrocytes in two isogenic donor lines (p= 0.003 and p < 0.0001, Fig. 3d). Consistent with this, immunoreactivity against the lysosomal-associated protein LAMP1 was significantly reduced in *APOE4/4* astrocytes compared to isogenic *APOE3/3* astrocytes (p = 0.0002 and p = 0.0001, Extended Data Fig. 3d). Likewise, staining with LysoSensor Green, a lysosomal dye that fluoresces most brightly at an acidic lysosomal pH of ~5.2, revealed that *APOE4/4* astrocytes have significantly decreased LysoSensor Green signal, indicating decreased lysosomal acidity compared to *APOE3/3* across isogenic *APOE3/3* and *APOE4/4 astrocytes* generated from 3 different individuals (p < 0.0001, p = 0.002, p = 0.0002, Extended Data Fig. 3e). These results suggest *APOE4/4* astrocytes have decreased LAMP1 positive lysosomes with decreased lysosomal acidity, contributing to impaired lysosomal proteolytic function.

Since phosphorylated α-Syn is used as a measurement of α-Syn pathology, we next determined whether astrocytes can phosphorylate neuronal α-Syn. Therefore, we exposed *APOE3/3* and *APOE4/4* astrocytes to fresh media or conditioned media from *SNCA*-A53T neurons. At baseline, *APOE4/4* astrocytes have more endogenous p-Syn compared to *APOE3/3* astrocytes (Fig. 3e; p < 0.0001). *APOE4/4* astrocytes incubated with media previously exposed to *SNCA*-A53T neurons for 3 days, showed a further increase in p-Syn (p = 0.007) while *APOE3/3* astrocytes were unaffected, (p = 0.232) (Fig. 3e) indicating that *APOE4/4* astrocytes can increase α-Syn phosphorylation. However, in the miBrain and post-mortem human brain, inclusions of α-Syn are primarily found inside neurons. We found that cell culture media collected from *SNCA*-A53T neurons and then inoculated onto fresh *SNCA*-A53T neuronal cultures itself does not increase phosphorylation of neuronal α-Syn (Fig. 3f), suggesting that non-cell-autonomous mechanisms likely modify α-synuclein’s pathogenicity. Therefore, we hypothesized that *APOE4/4* astrocytes fail to degrade α-Syn due to impaired endolysosomal function. Instead, *APOE4/4* astrocytes phosphorylate α-Syn and release the more pathogenic forms of a-Syn into the extracellular space allowing them to be taken up by neurons and promote the formation of neuronal α-Syn inclusions. To test this hypothesis, we first exposed *SNCA*-A53T neurons to conditioned media from only *APOE3/3* or *APOE4/4* astrocytes. Astrocyte-conditioned media itself did not induce significant p-Syn in *SNCA-A53T* neurons, and no significant difference was observed between *APOE3/3* and *APOE4/4* conditioned media (Extended Data Fig. 3f). We reasoned the low levels of α-Syn produced by astrocytes alone is not sufficient to induce phosphorylation and aggregation of neuronal α-Syn. Therefore, we performed a double conditioned media experiment where conditioned media was first collected from *SNCA*-A53T neurons and subsequently cultured with either *APOE3/3* or *APOE4/4* astrocytes. This double-conditioned media was then collected and inoculated onto fresh *SNCA*-A53T- neuron monocultures (Fig 3f). Neurons grown in *APOE3/3* double-conditioned media did not show a significant increase in α-Syn phosphorylation (p = 0.601). In contrast, *APOE4/4* double-conditioned media led to a 2.371-fold increase (± 0.315; SEM) in α-Syn phosphorylation (p = 0.0016). To assess whether the induction of p-Syn by *APOE4/4* double-conditioned media was a failure of astrocytes to degrade α-Syn or a modification of α-Syn, we analyzed the α-Syn species in the double-conditioned media via dot blot. Although the total levels of α-Syn in the media were not different between *APOE3/3* and *APOE4/4* astrocytes (p = 0.894), p-Syn and aggregated α-Syn were both significantly increased (p < 0.0001 and p = 0.0051) in media conditioned by *APOE4/4* astrocytes compared to *APOE3/3* astrocytes (Extended Data Fig. 3g). *APOE3/3* astrocyte double-conditioned media appeared not to change having similar p-Syn and aggregated α-Syn levels to the original *SNCA*-A53T media that was not conditioned by astrocytes (p = 0.951 and p = 0.894; Extended Data Fig. 3g). Collectively, these results suggest that *APOE4/4* astrocytes fail to degrade neuronal a-Syn, instead increase its phosphorylation and aggregation, and secrete these more pathological forms of α-Syn which are taken up by neurons and promote the formation of a-Syn inclusions.

### Cholesterol accumulation in APOE4 astrocytes leads to dysfunctional α-Syn processing and neuronal pathology.

**Extended Data Figure 4.**
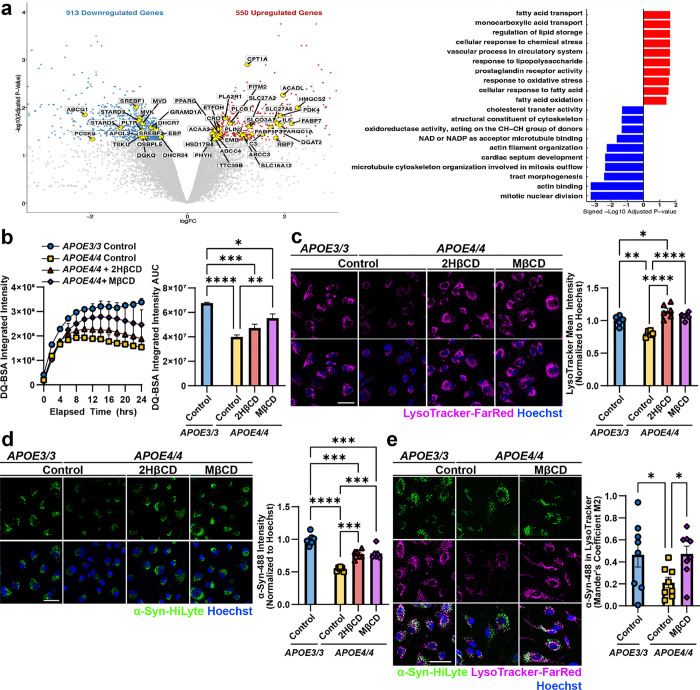
MβCD treatment in *APOE4/4* astrocytes improves lysosomal uptake of α-Synuclein. **a.** Volcano plot of *APOE4/4* vs. *APOE3/3* astrocytes showing DEGs (p val adj < 0.5, abs(logFC > 0.5). Top 10 Gene Ontology (GO) upregulated (red) and downregulated (blue) pathways ranked by adjusted p-value. Genes highlighted in the volcano plot correspond to GO terms related to lipid metabolism, including “fatty acid oxidation”, “cellular response to fatty acid”, “regulation of lipid storage”, “monocarboxylic acid transport”, “fatty acid transport”, “cholesterol transfer activity”, and “cholesterol metabolic process”. **b.** Left: DQ-BSA Red integrated intensity in astrocytes with 2HβCD or MβCD treatment, measured over 24 hours on an Incucyte (Sartorius) in a second isogenic donor line. Data points represent mean values and error bars represent standard error (n = 4 replicates). Right: Area under the curve calculation for DQ-BSA. Treatment of *APOE4/4* astrocytes with MβCD improved lysosomal proteolytic activity in a second donor line. Bars represent mean value and error bars represent standard error (n = 4 replicates). P-values were calculated by 2-way ANOVA followed by a Tukey test. **c.** Representative images of LysoTracker in astrocytes treated with cyclodextrins in a second isogenic donor line. Treatment with cyclodextrins increased endolysosomal area. Bars represent LysoTracker mean intensity (magenta) normalized by Hoechst area (blue). Error bars represent standard error (n = 5–6 replicates) P-values were calculated by 2-way ANOVA followed by a Sidak test. **d.** Representative images of astrocytes treated with cyclodextrins after a 24h incubation with fluorescently labeled α-Syn in a second isogenic donor line. Cyclodextrin treated astrocytes have increased α-Syn uptake compared to untreated *APOE4/4* astrocytes. Bars represent α-Syn mean intensity (green) normalized by Hoechst area (blue). Error bars represent standard error (n = 6 replicates) P-values were calculated by 2-way ANOVA followed by a Tukey test. **e.** Representative images of fluorescently labeled α-Syn co-localized with endolysosomes in astrocytes treated with cyclodextrins. Cyclodextrin treated astrocytes had increased co-localization between α-Syn and lysosomes compared to untreated *APOE4/4,* indicative of improved α-Syn uptake. Bars represent mean Mander’s coefficient M2 (amount of a-Syn signal in overlapping LysoTracker). Error bars represent standard error (n = 8 replicates). P-values were calculated by 2-way ANOVA followed by a Fisher’s LSD test. All scale bars = 50 μm. *P < 0.05, **P < 0.01, ***P < 0.001, ****P < 0.0001.

**Figure 4.**
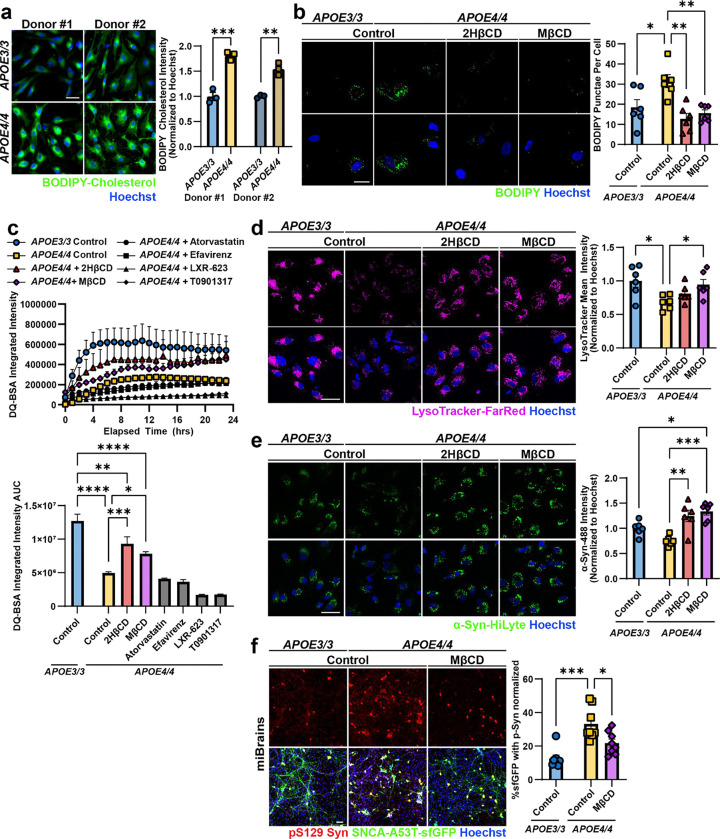
Reducing cholesterol improves *APOE4/4* astrocyte lysosomal and α-Syn homeostasis. **a.** Representative images of live astrocytes with BODIPY-cholesterol in two isogenic lines. *APOE4/4* astrocytes have significantly more BODIPY-cholesterol staining than *APOE3/3* astrocytes. Bars represent BODIPY-cholesterol mean intensity (green) normalized by Hoechst area (blue). Error bars represent standard error (n = 3 replicates). P-values were calculated by unpaired t-tests. **b.** Representative images of BODIPY staining in astrocytes treated with 2HβCD or MβCD. *APOE4/4* astrocytes have more BODIPY staining than *APOE3/3* astrocytes, which is reduced after treatment. Bars represent BODIPY punctae (green) per cell number (Hoechst; blue). Error bars represent standard error (n = 6 replicates). P-values were calculated by 2-way ANOVA followed by a Tukey test. **c.** Top: DQ-BSA Red integrated intensity, in astrocytes with 2HβCD, MβCD, atorvastatin, efavirenz, LXR-623, or T0901317 treatment, measured over 24 hours on an Incucyte (Sartorius). Data points represent mean values and error bars represent standard error (n = 4 replicates). Bottom: area under the curve calculation for DQ-BSA. Treatment of *APOE4/4* astrocytes with cyclodextrins, but not other drugs, improved lysosomal proteolytic activity. Bars represent mean value and error bars represent standard error (n = 4 replicates). P-values were calculated by 2-way ANOVA followed by a Tukey test. **d.** Representative images of LysoTracker in astrocytes treated with 2HβCD or MβCD. Treatment with cyclodextrins increased endolysosomal intensity. Bars represent LysoTracker mean intensity (magenta) normalized by Hoechst area (blue). Error bars represent standard error (n = 5–6 replicates) P-values were calculated by 2-way ANOVA followed by a Sidak test. **e.** Representative images of astrocytes treated with cyclodextrins after a 24h incubation with fluorescently labeled α-Syn, in two isogenic lines. Cyclodextrin treated astrocytes have increased α-Syn uptake compared to untreated *APOE4/4* astrocytes. Bars represent a-Syn mean intensity (green) normalized by Hoechst area (blue). Error bars represent standard error (n = 6 replicates) P-values were calculated by 2-way ANOVA followed by a Tukey test. **f.** Representative images of phosphorylated α-Syn in isogenic *APOE3/3* and *APOE4/4* miBrains. *APOE4/4* miBrains had significantly higher levels of pS129 (red) than APOE3/3. These levels were significantly reduced in *APOE4/4* miBrains treated with MβCD. Bars represent mean values of percent of sfGFP volume immunoreactive for phosphorylated α-Syn and error bars represent standard error (n = 8 replicates). P-values were calculated using two-way ANOVA followed by a Tukey test. All scale bars = 50 μm. *P < 0.05, **P < 0.01, ***P < 0.001, ****P < 0.0001.

We next investigated how APOE4 in astrocytes leads to lysosomal dysfunction and the spread of αSyn pathogenic forms. APOE is primarily expressed in astrocytes in the brain,^66^ shuttling cholesterol to neurons to maintain membranes and synapses^67,68^. APOE4 displays lower lipid transport affinity and binding capacity^69^, associated with cholesterol accumulation in astrocytic lysosomes and impaired lysosomal function^58,70^. Consistent with previous reports, transcriptomic analysis showed that genes in pathways associated with lipid storage and transport are differentially expressed compared to isogenic APOE3/3 astrocytes (Extended Data Fig. 4a)^58^, as well as response to lipopolysaccharide and oxidative stress, which have been associated with a neurotoxic reactive phenotype^71,72^. Consistent with a dysfunctional lipid metabolism, we found that *APOE4/4* astrocytes have a significant increase in the accumulation of BODIPY-cholesterol compared to isogenic *APOE3/3* astrocytes generated from two different individuals (p = 0.001 and p = 0.002, Fig. 4a).

Given our findings that *APOE4/4* astrocytes have increased cholesterol and impaired endolysosomal function, resulting in increased pathogenic α-Syn secretion, we hypothesized that intracellular accumulation of cholesterol leads to impaired degradation of α-Syn by astrocytes. To test this hypothesis, we treated *APOE4/4* astrocytes with various compounds to reduce cholesterol bioavailability, either by sequestering cholesterol (2-hydroxypropyl-β-cyclodextrin (2HβCD) and methyl-β-cyclodextrin (MβCD), inhibiting cholesterol biosynthesis (atorvastatin), or promoting cholesterol efflux (efavirenz, LXR-623, and T0901317). BODIPY staining confirmed a decrease in lipid droplets, which include cholesterol, in *APOE4/4* astrocytes following treatment with 2HβCD (p = 0.0013) and MβCD (p = 0.0063), to levels comparable to *APOE3/3* astrocytes (Fig. 4b). 2HβCD and MβCD significantly increased *APOE4/4* lysosomal proteolytic activity, as measured by the BSA-DQ assay, compared to vehicle treated *APOE4/4* astrocytes (2HβCD: p = 0.0005; MβCD: p = 0.046) (Fig. 4c). However, treatment with the other compounds had no effect, or a negative effect, on *APOE4/4* lysosomal proteolytic activity. We confirmed that MβCD increases lysosomal proteolytic activity in *APOE4/4* astrocytes generated from a second individual (p = 0.0093), although the effect of 2HβCD was not significant (p = 0.357; Extended Data Fig 4b). These findings suggest that improvement of proteolytic function may be selective to cyclodextrin treatments.

We further evaluated the effect of 2HβCD and MβCD on lysosomes by staining treated astrocytes with the live-cell endolysosomal dye, LysoTracker. *APOE4/4* astrocytes treated with MβCD had an increase in lysosomal staining intensity compared to control *APOE4/4* astrocytes (p = 0.044) (Fig. 4d). However, 2HβCD treatment did not have a significant effect on endolysosome intensity (p = 0.51). In astrocytes differentiated from a second individual, both cyclodextrin treatments significantly increased endolysosomal staining intensity in *APOE4/4* astrocytes (2HβCD: p < 0.0001; MβCD: p < 0.0001) to levels comparable to *APOE3/3* astrocytes (Extended Data Fig. 4c). These findings suggest that lowering cholesterol burden in *APOE4/4* astrocytes with 2HβCD or MβCD treatment is sufficient to increase lysosomal and proteolytic function in *APOE4/4* astrocytes.

To evaluate the effect of 2HβCD and MβCD treatment on α-Syn uptake and degradation, we measured α-Syn-HiLyte uptake after 24 hours in astrocytes. The intensity of intracellular α-Syn-HiLyte after 24 hours significantly increased in 2HβCD and MβCD treated *APOE4/4* astrocytes (2HβCD: p = 0.002; MβCD: p = 0.0003) compared to vehicle-treated *APOE4/4* astrocytes (Fig 4e). This effect was replicated in isogenic astrocytes from a second individual (2HβCD: p = 0.0004; MβCD: p = 0.0003) (Extended Data Fig 4d). To validate that the α-Syn was being endocytosed into the endolysosomal pathway, we incubated astrocytes with α-Syn-HiLyte for 4 hours and co-stained with LysoTracker. In agreement with our other findings that *APOE4/4* astrocytes have impaired α-Syn uptake, the percentage of α-Syn-HiLyte that co-localized with LysoTracker decreased in *APOE4/4* astrocytes compared to *APOE3/3* astrocytes (p = 0.0395; Extended Data Fig 4e). However, *APOE4/4* astrocytes treated with MβCD showed increased colocalization of α-Syn-HiLyte with LysoTracker compared to vehicle-treated *APOE4/4* astrocytes (p = 0.0343). Strikingly APOE4/4 astrocytes treated with MβCD had similar α-Syn lysosomal localization as vehicle-treated *APOE3/*3 astrocytes (p = 0.946; Extended Data Fig 4e). Taken together, these results suggest that pharmacologically reducing intracellular cholesterol in *APOE4/4* astrocytes increase α-Syn uptake and degradation potentially reducing the accumulation of neurotoxic forms of α-Syn.

Our findings implicate astrocytes as a critical cell type in α-Syn processing in the genetic context of *APOE4.* However, α-Syn pathology in Lewy body diseases is characterized by inclusions found within neuronal projections and soma. To investigate whether improved lysosomal activity in cyclodextrin-treated astrocytes affected neuronal α-Syn phosphorylation, we returned to the miBrain model. After 7 days of treatment with MβCD, the levels of neuronal phosphorylated α-Syn were significantly reduced in MβCD treated *APOE4/4* miBrains (p = 0.031) when compared to untreated *APOE4/4* miBrains and restored phosphorylation to levels that were not statistically different from *APOE3/3* miBrains (p = 0.099) (Fig. 4f). These results show that MβCD treatment reduces α-Syn phosphorylation in neurons likely via restoration of lysosomal activity and α-Syn processing in astrocytes, alleviating cells of the cytotoxic burden of α-Syn aggregation. This may open a therapeutic avenue for cholesterol-lowering pharmacological interventions in the treatment of synucleinopathies and AD with Lewy Bodies, particularly in *APOE4* carriers.

## Discussion:

By combining stem cell and genetic engineering, we extended our recently developed miBrain system^27^ to model α-Synuclein pathological phenotypes and dissect disease-relevant cellular and molecular mechanisms in human brain tissue in a dish. miBrains containing neurons, glia, and vascular cells displayed trackable α-Syn pathological forms in neurons and neuronal death as early as 2 weeks and as late as 6 months in culture. Cryopreservation of pre-assembled tissue allowed better reproducibility and decreased variability of our methods, enabling complex multivariate experiments. Using a genetic mix-and-match permutation approach that is unique to miBrains, we found that *APOE4* promotes neuronal α-Syn accumulation specifically via astrocytes. *APOE4*-induced cholesterol accumulation causes endolysosomal dysfunction and impaired α-Syn processing in astrocytes. This leads to the astrocytic secretion of pathogenic α-Syn that seeds α-Syn aggregation and phosphorylation on neurons. We found that pharmacologically increasing cholesterol efflux restored astrocytic endolysosomal function and prevented the accumulation of neuronal p-Syn in human brain tissue. These results establish a causal link between cholesterol dysregulation and α-Syn pathology in *APOE4* carriers, which may influence the progression of classical synucleinopathies and AD with Lewy Bodies.

Cholesterol dysregulation in *APOE4* astrocytes has been related to a reduced expression of lipid transport genes^57^, reduced cholesterol efflux^58^, and increased accumulation of cholesterol in lysosomes^70^. This was associated with increased secretion of inflammatory cytokines and reduced amyloid-β processing^73^. Genetically induced cholesterol accumulation has been shown to promote an increased secretion of exosomes^74^. Cholesterol accumulation might convert astrocytes into a secretory phenotype, including releasing pathogenic α-Syn and other cargo. While it is known that neurons can secrete pathogenic α-Syn^75^ that is taken and processed by astrocytes^61–63^, to our knowledge, the release of pathogenic α-Syn by dysfunctional astrocytes has not been previously described.

Our results suggest that *APOE4* leads to a disease-relevant astrocytic phenotype characterized by endolysosomal dysfunction driven by cholesterol accumulation, leading to suboptimal processing and secretion of pathogenic α-Syn that is further taken by neurons, exacerbating Lewy-like pathology. We found pharmacological cholesterol reduction using methyl-β-cyclodextrin prevented α-Syn accumulation in *APOE4* human brain tissue. Clinical trials on cyclodextrins have primarily focused on NPC1 disease, with a recent Phase I/II trial describing improved clinical symptoms and mild-to-moderate adverse effects after intravenous administration of hydroxypropyl-β-cyclodextrin^76^. Other cholesterol-lowering drugs such as statins remain the primary approach to reduce cholesterol in patients without NPC1. In a mouse model of α-Syn pathology, feeding a high-fat diet increased α-Syn aggregation and that was reduced by treatment with brain-penetrating statins, suggesting that statins can lower α-Syn aggregation by reducing brain cholesterol^77^. A meta-analysis study reported that statins can reduce the risk of Parkinsonism in older adults and that this effect is mediated by reduced atherosclerosis^78^, but a randomized clinical trial evaluating the benefit of the most used type of statin, simvastatin, found no evidence to support its use as a disease-modifying therapy in Parkinson’s disease^79^. We discovered that *APOE4* increases α-Syn pathology via cholesterol dysregulation, suggesting that the *APOE* genotype may impact whether cholesterol-lowering medications can benefit classic synucleinopathies and co-pathological presentations of pathogenic α-Syn such as AD with Lewy Bodies.

We present a novel, scalable stem cell-based tissue platform capable of modeling human neurodegenerative phenotypes in a dish. Collectively, our findings establish a mechanistic link between cholesterol accumulation and α-Syn pathology mediated by *APOE4* astrocytes and offer therapeutic opportunities for *APOE4* carriers with α-Syn pathology in classical synucleinopathies like LBD, as well as in AD with Lewy Bodies and other neurodegenerative conditions. Future optimization of the miBrain system to enable microvascular perfusion and support extended experimentation and drug testing will further enhance its utility, paving the way for deeper exploration of neurodegenerative mechanisms and therapeutic testing.

## Methods:

### Resource availability

#### Lead Contact

Further information and requests for resources and reagents should be directed to and will be fulfilled by the lead contact, Dr. Joel W. Blanchard (joel.blanchard@mssm.edu).

#### Material availability

This study did not generate new unique reagents

#### Data and code availability

- All raw data will be made available on Mendeley upon paper acceptance and will be made publicly available.
- Original code relating to scRNAseq pseudobulk analysis will be made available on Mendeley upon paper acceptance and will be publicly available.
- Any additional information required to reanalyze the data reported in this paper is available from the lead contact upon request.

#### Human iPSC cultures

All human iPSCs were maintained in feeder-free conditions in StemFlex^™^ medium (Gibco) on Geltrex^™^ Matrix (Thermo Fisher Scientific) pre-coated plates. All iPSC lines used in this study are listed in the Key Resource Table. CRISPR/Cas9 genome editing was performed as previously described^57^. Human iPSCs were grown as colonies in StemFlex^™^ medium until they reached 60–70% confluency. At this point, iPSCs were either passaged for maintenance using 0.5 mM EDTA to gently lift colonies or harvested using Accutase^™^ cell detachment solution for 5–10 minutes at 37C to start a differentiation protocol as singularized cells.

#### Differentiation of human iPSCs into neurons

Neuron differentiation was adapted from Zhang *et al*.^30^ and Lam *et al*.^29^. Briefly, iPSCs were transfected with PiggyBac plasmids to confer doxycycline-inducible expression of the Neurogenin- 2 gene (*NGN2*, Addgene Plasmid #209077) alone or combination with SNCA-A53T-sfGFP (Addgene Plasmid: 209080) or A53T-DNAC-SNCA-sfGFP (Addgene Plasmid: 209081), using Lipofectamine^™^ Stem Transfection Reagent. Briefly, dissociated iPSCs were plated at ~104,000 cells/cm^2^ onto Geltrex^™^-coated plates, in StemFlex^™^ supplemented with 10 μM Y27632 and 5 μg/mL doxycycline (day 0). At day 1, medium was replaced with Neurobasal N2B27 medium (Neurobasal, 1x B-27, 1x N-2, 1x MEM-NEAA, 1x GlutaMAX, 1% penicillin-streptomycin) supplemented with 10uM SB431542, 100 nM LDN, 5 μg/mL doxycycline, 5 μg/mL Blasticidin. On day 2, the medium was replaced with Neurobasal N2B27 media supplemented with 10uM SB431542, 100 nM LDN, 5 μg/mL doxycycline, 1 μg/mL puromycin. On days 3–6, the medium was replaced daily with Neurobasal N2B27 media supplemented with 5 μg/mL doxycycline, and 1 μg/mL puromycin. At day 7, cells were dissociated with Accutase and either seeded into miBrains (see bellow) or seeded into 2D monocultures on Poly-L-Ornithine and Laminin pre-coated plates at 156,250 cells/cm^2^, in Neurobasal N2B27 with 5 μg/mL doxycycline and 10 μM Y27632. For 2D cultures, on day 8, wells were gently topped with Neurobasal N2B27 supplemented 20ng/mL BDNF, 20ng/mL GDNF, 1mM dcAMP, 2ug/mL Laminin, and 1 uM AraC, using the same volume of medium in the wells. At day 11, media is replaced with Neurobasal N2B27 supplemented with 10ng/mL BDNF, 10ng/mL GDNF, 0.5 mM dcAMP, 1ug/mL Laminin. This medium was used for half-media changes every 3–4 days.

#### Differentiation of human iPSCs into astrocytes

Astrocytes were generated using previously published protocols for iPSC-derived NPC (Chambers *et al*.)^31^ and astrocyte (TCW *et al*.)^32^ differentiation. Briefly, dissociated iPSCs were plated at 100,000 cells/cm^2^ onto Geltrex^™^-coated plates, in pre-warmed StemFlex^™^ supplemented with 10 μM Y27632. Cells were fed every other day with StemFlex^™^ until they reached >95% confluence. Once cells reached confluence, the medium was replaced with NPC medium (1:1 DMEM/F12: Neurobasal Medium, 1x N-2 Supplement, 1x B-27 Serum-Free supplement, 1x GlutaMAX Supplement, 1x MEM-NEAA, 1% penicillin-streptomycin) supplemented with 10 μM SB43152 and 100 nM LDN193189 (day 0). From days 1 to 9, cells are fed daily with NPC medium plus 10 μM SB43152 and 100 nM LDN193189. At day 10, cells were split with Accutase and replated onto fresh Geltrex^™^-coated plates, in NPC media supplemented with 20 ng/mL bFGF and 10 μM Y27632. From days 11 to 13, cells were fed with NPC media plus 20 ng/mL bFGF. At day 14, cells were split with Accutase and re-seed onto fresh Geltrex^™^-coated plates, in NPC media plus 20 ng/mL bFGF and 10 μM Y27632. Starting from day 15, cells were fed every 2–3 days with Astrocyte Medium (AM, ScienCell) and passaged using Accutase once they reached 90% confluence. From this point, NPCs were fully differentiated into astrocytes in 30 days. NPCs and fully differentiated astrocytes were cryopreserved in the freezing medium consisting of 90% knockout serum replacement (KSR) and 10% dimethyl sulfoxide (DMSO).

#### Differentiation of human iPSC into brain microvascular endothelial cells

Brain endothelial cell differentiation was adapted from the protocols from Blanchard *et al.*, Qian *et al.,* and Wang *et al*. ^33,36,80^. Briefly, iPSCs were transfected with a PiggyBac plasmid to confer doxycycline-inducible expression of the ETS variant transcription factor 2 (*ETV2*, Addgene Plasmid #168805), using Lipofectamine^™^ Stem Transfection Reagent. Inducible ETV2-iPSCs were grown until 60–70% confluency, dissociated with Accutase, and plated at 20,800 cells/cm^2^ onto Geltrex^™^-coated plates in StemFlex^™^ supplemented with 10 μM Y27632 (day 0). On day 1, the medium was replaced with DeSR1 medium (DMEM/F12 with GlutaMAX, 1× MEM-NEAA, 1× penicillin-streptomycin) supplemented with 10 ng/mL BMP4, 6 μM CHIR99021, and 5 μg/mL doxycycline. On day 3, the medium was replaced with DeSR2 medium (DeSR1 media, 1x N-2, 1× B-27) supplemented with 5 μg/mL doxycycline. At days 5 and 7, the medium was replaced with hECSR medium (Human Endothelial Serum-free Medium, Gibco, 1× MEM-NEAA, 1× B-27, 1% penicillin-streptomycin) supplemented with 50 ng/mL VEGF-A, 2 μM Forskolin, and 5 μg/mL doxycycline. At day 8, cells were dissociated using Accutase and re-seed onto fresh Geltrex^™^-coated plates in hECSR supplemented with 50 ng/mL VEGF-A and 5 μg/mL doxycycline. This medium was used for every 2–3 days medium change to maintain cells for up to 1 week until ready for tissue assembly (miBrain or JAMs).

#### Differentiation of human iPSCs into mural cells

Mural cells were differentiated using previously published protocol from Patsch *et al.*^37^. Dissociated iPSCs were plated at 37,000 to 40,000 cells/cm^2^ onto Geltrex^™^-coated plates, in StemFlex^™^ supplemented with 10 μM Y27632 (day 0). On day 1, the medium was replaced with N2B27 medium (1:1 DMEM/F12: Neurobasal media, 1x B-27, 1x N-2, 1x MEM-NEAA, 1x GlutaMAX, 1% penicillin-streptomycin) supplemented with 25 ng/mL BMP4 and 8 μM CHIR99021. At days 3 and 4, the medium was replaced with N2B27 media supplemented with 10 ng/mL Activin A and 10 ng/mL PDGF-BB. At day 5, mural cells were dissociated with Accutase and re-seeded onto fresh 0.1% gelatin-coated plates at 35,000 cells/cm^2^, in N2B27 supplemented with 10 ng/mL PDGF-BB. This medium was used every 2–3 days medium was change for another 5–7 days. Cells were then banked in freezing medium (90% KSR/ 10% DMSO) and expanded in N2B27 until ready for tissue assembly (miBrain or JAMs).

#### Differentiation of human iPSCs into oligodendrocyte progenitor cells (OPCs)

OPC differentiation was adapted from Douvaras et al, 2014^81^. Briefly, iPSCs were dissociated into single cells using Accutase and seeded at near-confluent density. Differentiation began the next day (designated as day 0) by culturing the cells in DMEM/F12 (1:1) medium supplemented with N2, 10 μM SB431542, 100 nM LDN 193189, and 100 nM all-trans retinoic acid (RA), with daily medium changes. On day 8, 1 μM SAG was added to the differentiation medium, maintaining the presence of 10 μM SB431542 and 100 nM LDN 193189. By day 12, adherent cells were detached and transferred to low-attachment plates to form cell spheres. These spheres were cultured in DMEM/F12 (1:1) medium containing N2, RA, and SAG. On day 30, spheres were plated onto poly-L-ornithine/laminin-coated plates to allow cells to migrate outward. At this stage, the medium was replaced with DMEM/F12 (1:1) supplemented with N2, B27, 10 ng/ml PDGF-AA, 10 ng/ml IGF, 5 ng/ml HGF, 10 ng/ml NT3, 25 μg/ml insulin, 100 ng/ml biotin, 1 μM cAMP, and 60 ng/ml T3. By day 75, cells were harvested, dissociated, and purified using NG2-specific magnetic-activated cell sorting (MACS). The enriched cells were expanded in DMEM/F12 (1:1) medium supplemented with N2, B27 without Vitamin A, 10 ng/ml PDGF-AA, 10 ng/ml β-FGF, and 10 ng/ml NT3 until ready for tissue assembly (miBrain or JAMs).

#### Differentiation of human iPSCs into microglia

iPSC-derived microglia were differentiated as previously shown^39,82^ via an intermediate differentiation step into hematopoietic progenitor cells (HPCs). For the generation of HPCs, the STEMdiff Hematopoietic Kit (cat#: 05310; StemCell Technologies) was used, according to the manufacturer’s manual. Briefly, when 70% confluent (day 0), iPSCs were harvested and passaged at a density of 20–40 colonies per well in a 6-well coated with 0.1mg/mL Matrigel (cat#: 354234; Corning). On day 1, Medium A was added to the culture, and on day 4 it was switched to Medium B until complete HPC maturation on days 11–13. Fully differentiated HPCs, floating in the medium and detached from the colonies, were collected for microglial differentiation or frozen in Stem-CellBanker (cat#: 11924; AMSBIO) supplemented with microglial cytokines. For the generation of mature microglia, differentiated HPCs were collected and transferred into Matrigel coated 6-well plate at a confluency of 350k HPCs per well. The differentiation takes 25–28 days, during which HPCs are cultured in microglia media consisting of DMEM/F12 (cat#: 11320–033; Gibco), with 2X B27 (cat#: 17504044; Thermo Fisher Scientific), 0.5X N2 (cat#: 17502048; Thermo Fisher Scientific), 1X Glutamax (cat#: 35050061; Gibco), 1X non-essential amino acids (cat#: 11–140-050; Gibco, 400 mM Monothioglycerol (cat#: M6145; Millipore Sigma), and 5 mg/mL human insulin (cat#: I9278; Millipore Sigma), freshly supplemented with 100 ng/mL IL-34 (cat#: 200–34; PeproTech), 50 ng/mL TGFβ1 (cat#: 100–21; PeproTech), and 25 ng/mL M-CSF (cat#: 300–25; PeproTech). Microglia were added to miBrains within the pool of Geltrex encapsulated cells at the time of miBrain assembly. MiBrains were maintained in miBrain media supplemented with 100 ng/mL IL-34 and 25 ng/mL M-CSF for 1 week and then switched to miBrain media supplemented with 25 ng/mL M-CSF until downstream experiments.

#### 3D Tissue Assembly for miBrains, co-cultures or Cryopreservation

Neurons, astrocytes, endothelial cells, mural cells, and OPCs were dissociated using Accutase or TryplE Select (astrocytes). Cells were resuspended in corresponding media, counted, and resuspended at 1 × 10^6^ cells/ mL. For miBrains, a tube was prepared to contain 5 × 10^6^ neurons, 5 × 10^6^ endothelial cells, 1 × 10^6^ astrocytes, 1 × 10^6^ OPCs, and 1 × 10^6^ mural cells per 1 mL. Microglia was added for a subset of miBrains at the ratio of 1.67 × 10^6^ per 1 mL. Pooled cells were spun down at 200 × g for 5 min at RT. Media was aspirated carefully, leaving the cell pellet undisturbed. The cell pellet was placed on ice and resuspended in 1 mL Geltrex^™^ supplemented with 10 μM Y27632 and 5 μg/mL doxycycline, avoiding air bubbles and keeping it on ice to prevent premature Geltrex polymerization and inability to seed miBrains properly. To generate miBrain tissue that adopted a free-floating, organoid-like morphology over time, 25–50 μL of encapsulated cell suspension were seeded per inner glass-bottom well of a 48-well MatTek plate (MatTek). To generate miBrain tissue that remained attached to the plate (more suitable for automated imaging) while conserving 3D morphology, 10 μL of encapsulated cell suspension were seeded per well of a 96-well μClear plastic-bottom plate (Greiner). For JAMs assembly and cryopreservation, a tube containing 5 × 10^6^ endothelial cells, 1 × 10^6^ astrocytes, 1 × 10^6^ OPCs, and 1 × 10^6^ mural cells per 1 mL was prepared. Pooled cells were spun down at 200 × g for 5 min at RT and cryopreserved in miBrain freezing media (60% KSR, 30% hECSR medium, 10% DMSO, 10 μM Y27632, 50 ng/mL VEGF-A). Upon thaw, cell viability was assessed, and the appropriate volume of neurons needed to conserve the original miBrain cell-to-cell ratio was added to the pooled cell suspension, which was spun again, encapsulated, and seeded as described above. After miBrains were seeded, the plates were transferred into a 37 °C 95%/5% Air/CO2 incubator for 30 minutes to allow the Geltrex^™^ to polymerize. After polymerization of the gel, miBrain week-1 medium (Human Endothelial Serum-free Medium, 1x Pen/Strep, 1X MEM-NEAA, 1X CD Lipids, 1x Astrocyte Growth Supplement (ScienCell), 1x B27 Supplement, 10ug/mL Insulin, 1 μM cAMP-dibutyl, 50 μg/mL Ascorbic acid, 10ng/mL NT3, 10ng/mL IGF, 100ng/mL Biotin, 60 ng/mL T3, 50 ng/mL VEGF, 1 μM SAG, 5 μg/mL doxycycline) was added to each well, ensuring complete submersion of the culture in media (250–500uL per well of a 48-well matTek plate, 100 to 200 uL per well of a 96-well plate). Half media change was performed every 2–3 days. On day 8 after miBrain seeding, the media was changed to miBrain week-2 medium (Human Endothelial Serum-free Medium, 1x Pen/Strep, 1X MEM-NEAA, 1X CD Lipids, 1x Astrocyte Growth Supplement (ScienCell), 1x B27 Supplement, 10ug/mL Insulin, 1 μM cAMP-dibutyl, 50 μg/mL Ascorbic acid, 10ng/mL NT3, 10ng/mL IGF, 100ng/mL Biotin, 60 ng/mL T3, 5 μg/mL doxycycline). Cultures were used for downstream assays after 2 weeks.

#### Exposure of tissue to exogenous α-Synuclein

miBrains were exposed to 4 mg/ml of Human Recombinant Alpha Synuclein Protein Aggregates (Pre Formed Fibrils, PFFs, from StressMarq, #SPR-322, or Abcam, #ab218819). PFFs were sonicated in a water bath (VWR Ultrasonic Cleaner) immediately before adding to the cultures, using 10 cycles of 30 sec on, and 30 sec off. Free-floating miBrains incubated with Alpha-Synuclein had a media change after 48h and were fixed in 4% paraformaldehyde after 2 weeks.

#### Induction of α-Syn pathology via expression of SNCA-A53T

Neurons with inducible expression of *SNCA* with the A53>T mutation (A53T), which increases α-Syn’s propensity to aggregate^44,45^, were generated from iPSC as described above. Cells were harvested on day 7 of the differentiation and cultured in 2D on Poly-L-Ornithine and Laminin pre-coated plates at 156,250 cells/cm^2^, or encapsulated for 3D cultures in Geltrex at 50,000 cells for 10 uL of Geltrex, or added to JAMs for miBrain assembly as described above. The cultures were maintained for 18 days with or without PFFs. For the cultures that received PFFs, 4 mg/ml PFFs were added to the media on day 4 after assembly. The media was half-changed every 2–3 days. As an additional control, we generated neurons where SNCA-A53T was overexpressed without the non-amyloid component (NAC) domain, which is required for α-Syn aggregation.^83^

#### Immunofluorescence

2D cultures were fixed in 4% paraformaldehyde for 15 min at room temperature and rinsed with PBS. 2D cultures were blocked in 0.3% Triton-X100, 5% normal donkey serum in PBS for 30 min., and then with primary antibodies diluted in blocking buffer overnight at 4°C. Cultures were rinsed 3 times with 0.3% Triton-X100 in PBS for 15 min each, and incubated with secondary antibodies (and Hoechst 33342) diluted at 1:1000 in blocking buffer for 2h at room temperature. Cultures were rinsed 3 times with PBS for 15 minutes and left in PBS for image acquisition. 2D neuronal cultures were blocked in 0.1% Triton-X100, and 10% normal donkey serum in PBS, antibodies were diluted in 0.02% Triton-X100 and 2% normal donkey serum in PBS, and washes were done with PBS. All other conditions were kept the same as the other cell types.

3D cultures and miBrains were fixed in 4% paraformaldehyde overnight at 4°C and rinsed with PBS. 3D cultures and miBrains were incubated in blocking solution (0.3% Triton-X100, 5% normal donkey serum, in PBS) overnight at 4°C and then with primary antibodies diluted at 1:500 in blocking solution for 2–3 nights at 4°C. Cultures were rinsed 5 times with 0.3% Triton-X100 PBS for 30 min each and incubated with secondary antibodies and Hoechst 33342 (Thermo 62249) diluted at 1:1000 in blocking solution for 2–3 nights at 4°C. Cultures were rinsed 5 times with 0. 3% Triton-X100 PBS for 30 min each then rinsed and left in PBS for image acquisition.

#### Image acquisition and quantification

Images were acquired using a confocal microscope (Leica Stellaris or Nikon AX R). For quantification, 10–20 μm Z-stack images were acquired at 10x or 20x, 4 fields per well or free-floating miBrains. Volume measurements on Z-stacks were performed using Nikon AX R built-in quantification software. Statistical analyses were performed using GraphPad Prism Software. Normality and Lognormality tests D’Agostino & Pearson, Anderson-Darling, Shapiro-Wilk and Kolmogorov-Smirnov were performed to determine the choice for parametric or non-parametric testing. Post hoc tests on ANOVAs were conducted based on GraphPad reccomendations.

#### Human iPSC-derived astrocyte bulk RNAseq analysis

Fragments Per Kilobase of transcript per Million mapped reads (FPKM) values were obtained from published bulk RNA-seq data of isogenic human iPSC-derived astrocytes expressing either *APOE3/3* or *APOE4/4*^57^. FPKM values were log2-transformed with an offset of 0.1 and genes with zero variance across all the samples were excluded. To focus on genes with meaningful variability, an additional filtering step was applied to retain genes above the 10th percentile (variance > 0.033) after the removal of the zero-variance genes. Differential expression analysis was performed using the limma package in R. A design matrix was constructed to model the experimental conditions (APOE3 vs. APOE4) and sample-specific array weights were estimated using the array Weights function with a prior.n value of 100 to help stabilize weight estimation. A linear model was then fit to the log-transformed, filtered expression data using lmFit, and empirical Bayes moderation was applied via the eBayes function, with trend and robust both set to TRUE. Differentially expressed genes (DEGs) were defined as having an absolute log2 fold change > 0.5 and adjusted p-value (false discovery weight) < 0.05. A volcano plot was then generated using the ggplot2 package and genes meeting the DEG thresholds were highlighted. Enrichment analysis was performed on the upregulated and downregulated DEGs separately using the clusterProfiler package. Pathways were identified through Gene Ontology (GO) database. The top 10 pathways for each direction (upregulated and downregulated) were selected based on adjusted p-values.

#### Human astrocyte scRNAseq pseudobulk analysis

The processed dataset was downloaded from Haney et al^64^. Unless otherwise stated, all following analyses were performed in R using the package Seurat^84^. Aligning with the quality control protocol from the source publication, cells with nFeature < 500, nCount < 1000, and mitochondrial and ribosomal reads > 10% were discarded. Doublets were removed using the DoubletFinder package^85^. Following the standard Seurat pipeline with default parameters, raw gene counts were normalized, the top 2,500 highly variable genes were identified, and the data was scaled. Harmony was used to integrate the patient samples, and the top 20 principal components were used in Seurat’s FindNeighbors, FindClusters (0.2 resolution), and RunUMAP functions. Cell-type annotation was manually performed using the marker genes described in Haney et al. and clusters not enriched for these marker genes were removed from further analysis. Pseudobulked samples were generated by summing the raw counts for each patient sample and normalized using Seurat’s AggregateExpression() function.

#### Isogenic iPSC-derived astrocyte RNAseq analysis

The raw counts from iPSC-derived astrocyte bulk RNAseq were downloaded from Lin et al.^57^ Raw counts were processed using the DeSeq2 package^86^ and normalized using the counts() function with normalized set to TRUE.

#### DQ-BSA proteolytic activity assay

Astrocytes were seeded at 10,000 cells/well of a 96 well μClear plastic bottom plate (Greiner 655090) in AM. The following day, media was changed to FBS-free maturation media (50% DMEM/F12, 50% Neurobasal, 1X B27 without Vitamin A, 1X N2, 1X NEAA, 1X GlutaMAX, 1% penicillin-streptomycin). Half of the wells were treated with 100 nM BafilomycinA1 to inhibit lysosomal function as a negative control. The next day, cells were pulsed with 1mM DQ-BSA (Thermo D12051) in maturation media for 30 minutes. Media was replaced with fresh media (and fresh bafilomycin in the appropriate wells), and the red fluorescence and bright field were imaged at 20x with an Incucyte (Sartorius) every hour for 24 hours.

Alternatively, astrocytes were seeded at 50K cells/well of a 12-well plate. Media changes were followed as above. Cells were lifted to single-cell suspension with TrypLE Select 24 hours after the DQ-BSA pulse and filtered into flow cytometry tubes with DAPI. Red fluorescence in live cells was analyzed with a BD Celesta flow cytometer.

#### LysoSensor by flow cytometry

Astrocytes were seeded at 50,000 cells/well of a 12-well plate in AM. The following day, media was changed to FBS-free maturation media (50% DMEM/F12, 50% Neurobasal, 1X B27 without Vitamin A, 1X N2, 1X NEAA, 1X GlutaMAX, 1% penicillin-streptomycin), and cultured for 3 more days. Cells were incubated in 100 nM LysoSensor Green DND-189 (Thermo L7535) for 1 minute. Cells were washed and lifted to single cells suspension with media changes followed as above. Cells were lifted to single-cell suspension with TrypLE Select and filtered into flow cytometry tubes with DAPI. Green fluorescence in live cells was analyzed with a BD Celesta flow cytometer.

#### Live Imaging of α-Syn-HiLyte Uptake and Degradation

Astrocytes were seeded 10,000 cells/well of a 96 well μClear plastic bottom plate (Greiner) in AM. The following day, the media was changed to FBS-free maturation media (50% DMEM/F12, 50% Neurobasal, 1X B27 without Vitamin A, 1X N2, 1X NEAA, 1X GlutaMAX, 1% penicillin-streptomycin), and cultured for 2 more days. α-Syn HiLyte (AnaSpec AS-55457) was added to the media at a final concentration of 2 μg/mL and nuclei were stained with 10 μg/mL Hoechst 33342. Images were acquired every 30–90 minutes for the first 6 hours on a Nikon AX R. At 24 h, the media was changed to fresh FBS-free maturation media to remove any excess α-Syn. Images were acquired at 24 h and 48 h using the same parameters and laser settings.

#### Live Imaging of BODIPY-Cholesterol

Astrocytes were seeded 10,000 cells/well of a 96 well μClear plastic bottom plate (Greiner) in AM. The following day, the media was changed to FBS-free maturation media (50% DMEM/F12, 50% Neurobasal, 1X B27 without Vitamin A, 1X N2, 1X NEAA, 1X GlutaMAX, 1% penicillin-streptomycin), and cultured for 3 more days. Astrocytes were incubated with 2 μM BODIPY-Cholesterol (Cayman 24618) for 2 hours. Cells were then incubated with 10 μg/mL Hoechst 33342 for 10 minutes to stain nuclei and then washed. Media was replaced with fresh media and cells were imaged using a CX7 High Content Screening Platform with a 20x objective lens (Thermo; LUCPLFLN20x).

#### Western blotting and dot blotting

For Western blots, the protein concentration of each sample was measured using Pierce BCA Protein Assay (Thermo Fisher). Volumes corresponding to 20 μg of protein for each sample were loaded into Criterion Precast gels (BioRad), and a current of 120V was applied for approximately 60 min. The gel proteins were transferred to a PVDF membrane using BioRad TransBlot Turbo Transfer System, fixed in 4% PFA for 40 min and blocked in 5% w/v non-fat milk in 0.1% Tween in TBS (TBST) for 1 hour. For antibodies against phosphorylated proteins, blots were blocked in 5% w/v bovine serum albumin (BSA) in 0.1% TBST. Blots were then incubated with primary antibodies diluted at 1:1000 in blocking buffer overnight at 4°C. Membranes were washed in 0.1% TBST 3 times for 5 min each, incubated with secondary antibodies conjugates with horseradish peroxidase for 2h at room temperature in blocking buffer, and exposed to chemiluminescence activator before imaging using LI-COR Odyssey XF system. For dot blots, 3 mg of cell protein lysate or 3 ml of media were added onto nitrocellulose membranes and allowed to dry. Blocking and antibody incubation was performed as described for Western blots.

#### Collection and treatment with conditioned media

To generate neuron-conditioned media neurons were plated at ~150,000/cm2 on poly-L-ornithine and laminin coated plates on day 7 of differentiation. Media was changed following the differentiation protocol described above. Starting day 14 of differentiation, the media was collected, centrifuged at 2000xg for 5 minutes to pellet debris, and stored at −20°C. Media was collected with each half media change, every 3–4 days.

To generate astrocyte conditioned media, astrocytes were plated at ~30,000/cm2 on 0.1% gelatin-coated plates in AM. The following day, media was changed to either fresh neuron media (Neurobasal, 1x B-27, 1x N-2, 1x MEM-NEAA, 1x GlutaMAX, 1% penicillin-streptomycin, 10ng/mL BDNF, 10ng/mL GDNF, 0.5 mM dcAMP, 1ug/mL Laminin) supplemented with 10ng/mL CNTF or neuron conditioned media supplemented with 10ng/mL CNTF. Media was collected, centrifuged at 2000xg for 5 minutes to pellet debris, and stored at −20°C after 3 days.

To treat neurons with conditioned media, SNCA-A53T overexpressing neurons were plated at ~150,000/cm2 on poly-L-ornithine and laminin-coated 96-well μClear plates (Greiner) on day 7 of differentiation, following the normal neuron differentiation protocol described above. On day 11 of differentiation, the media was changed to conditioned media supplemented with 5 μg/mL doxycycline and 1 μg/mL laminin. Half media changes with conditioned media supplemented with doxycycline and laminin were continued every 3–4 days until day 25 of differentiation when cultures were fixed.

#### Treatment with cholesterol-lowering drugs

Astrocytes were seeded into 0.1% gelatin-coated 6 well plates in AM (ScienCell). After 2–3 days, the media was changed to AM without FBS supplemented with cholesterol-lowering drugs or DMSO as a vehicle. After 4 days, cells were seeded for final assays in 0.1% gelatin-coated 96-well μClear plates (Greiner) in AM with treatment. The following day, media was changed to FBS-free maturation media (50% DMEM/F12, 50% Neurobasal, 1X B27 without Vitamin A, 1X N2, 1X NEAA, 1X GlutaMAX, 1% penicillin-streptomycin) with treatment. Assays were started 2–3 days later, as described above. miBrains were treated with methyl-β-cyclodextrin (concentration below) or DMSO from day 0 of assembly to day 18, with half-media changes performed 3 times a week.

The following drug concentrations were used:

2-hydroxypropyl-β-cyclodextrin: 100 μM (Sigma C0926)

methyl-β-cyclodextrin: 100 μM (Cayman 21633)

atorvastatin: 50 nM (Sigma SML3030)

efavirenz: 10 μM (MedChemExpress HY10572)

LXR-623: 10 μM (MedChemExpress HY-10629)

T0901317: 10 μM (MedChemExpress HY-10626)

## Extended Data

## Supplementary Material

Supplement 1 **Extended Data Video 1.** Neurons with α-synuclein pathological phenotypes within the miBrain. 3D rendering with animated orthogonal projection of SNCA-A53T neurons within the miBrain. Green: SNCA-A53T-sfGFP; red: pS129 α-Syn; magenta: TUJ1, blue: Hoechst.

Supplement 2 **Extended Data Video 2. APOE3/3 astrocytes co-localize with neuronal-derived α-synuclein.** 3D rendering with animated orthogonal projection of astrocytes within an *APOE3/3* miBrain. Astrocytes display elongated morphology along neuronal networks. Green: SNCA-A53T-sfGFP; magenta: GFAP, blue: Hoechst.

Supplement 3 **Extended Data Video 3. *APOE4/4* reactive astrocytes poorly co-localize with neuronal-derived α-synuclein.** 3D rendering with animated orthogonal projection of astrocytes within an *APOE4/4* miBrain. Astrocytes display amoeboid morphology not aligned with neuronal networks. Green: SNCA-A53T-sfGFP; magenta: GFAP, blue: Hoechst.

## Figures and Tables

**Key Resources Table:** 

REAGENT OR RESOURCE	SOURCE	IDENTIFIER
**Chemicals, peptides, and recombinant proteins**		
2-hydroxypropyl-β-cyclodextrin	Sigma	Cat# C0926
Accutase	Stemcell	Cat# 07920
Activin A	Peprotech	Cat# 120-14P
Astrocyte Growth Supplement	ScienCell	Cat#1852
Astrocyte Medium (AM)	ScienCell	Cat# 1801
a-Synuclein (1-140) HiLyte Fluor 488	Anaspec	Cat# AS-55457
Atorvastatin	Sigma	Cat# SML3030
B27	Gibco	Cat# 17504044
B27 without Vitamin A	Gibco	Cat# 12587010
Bafilomycin A1	Millipore	Cat# B1793
BDNF	Peprotech	Cat# 450-02
Biotin	Sigma	Cat#B4639
Blasticidin	Gibco	Cat # A1113903
BMP4	Peprotech	Cat# 120-05ET
BODIPY-Cholesterol	Cayman	Cat# 24618
CD Lipids	Gibco	Cat# 11905031
CHIR99021	Tocris	Cat# 4423
CNTF	Peprotech	Cat# 450-13
DAPT	Cayman	Cat# 13197
Dibutyryl cAMP	Biogems	Cat# 1698950
DMEM	Gibco	Cat# 11965092
DMEM/F12 with GlutaMAX	Gibco	Cat# 10565018
Doxycyline	Millipore	Cat# D3072
DQ-BSA Red	Thermo Scientific	Cat # D12051
DRAQ5	Thermo Scientific	Cat# 52251
Efavirenz	MedChem Express	Cat# HY10572
FGF-basic	Peprotech	Cat# 100-18B
Forkskolin	R&D	Cat# 1099/10
GDNF	Peprotech	Cat# 450-10
Gelxtrex	Gibco	Cat# A1413201
GlutaMAX	Gibco	Cat# 35050061
HGF	Peprotech	Cat# 100-39H
Hoechst33342	Thermo Scientific	Cat# 62249
Human Endothelial SFM	Gibco	Cat# 11111044
IGF-1	Peprotech	Cat# 100-11
Insulin	Sigma	Cat# I9278
Laminin	Gibco	Cat# 23017015
L-Ascorbic Acid	Fisher Scientific	Cat# BP351
LipidSpot	Biotium	Cat# 70069-T
LDN193189	Tocris	Cat# 6053
Lipofectamine Stem Transfection Reagent	Invitrogen	Cat# STEM00001
LXR-623	MedChem Express	Cat# HY-10629
LysoSensor Green DND-189	Thermo Scientific	Cat# L7535
LysoTracker DeepRed	Thermo Scientific	Cat# L12492
MEM-Non-Essential Amino Acids	Gibco	Cat#111400050
Methyl-β-cyclodextrin	Cayman	Cat #21633
N2	Gibco	Cat# 17502048
Neurobasal	Gibco	Cat# 21103049
NT3	Peprotech	Cat# 450-03
PDGF-AA	Peprotech	Cat# 100-13A
PDGF-BB	Peprotech	Cat# AF-100-14B
Penicillin-Streptomycin	Gibco	Cat# 15140122
Puromycin	Gibco	Cat# A1113803
Retinoic Acid	MIllipore	Cat# R2625
SAG	Cayman	Cat# 11914
SB431542	Stemgent	Cat# 04-0010
StemFlex	Gibco	Cat# A3349401
T0901317	MedChem Express	Cat# HY-10626
TrypLE Select	Gibco	Cat# 12563011
VEGF-A	Peprotech	Cat#100-20
Y-27632	Tocris	Cat# 1254
**Antibody**		
a-Synuclein (MJFR1)	Abcam	Cat# ab138501
a-Synuclein (phospoho S129) (MJF-R13)	Abcam	Cat# ab168381
a-Synuclein pS129 (D1R1R)	CellSignaling	Cat# 23706
a-Synuclein pS129 (EP1536Y)	Abcam	Cat# ab51253
aSynuclein aggregate (MJFR-14-6-4-2)	Abcam	Cat# ab209538
AQP4	Invitrogen	Cat# PA5-53234
CD31/PECAM-1	R&D Systems	Cat# AF806
CD49f	BioLegend	Cat# 313602
GFAP	Invitrogen	Cat# 14-9892-82
IBA1	NovusBio	Cat# NB100-1028
LAMP1	NovusBio	Cat# NBP2-25183
MAP2	Biolegend	Cat# 822501
MBP	EMD Millipore	Cat# AB9348
MRF	EMD Millipore	Cat# ABN45
Neurofilament-H	BioLegend	Cat# 822601
NG2	EMD Millipore	Cat# Ab5320
S100β	Sigma-Aldrich	Cat# 2532
Tom20 (F-10)	Santa Cruz Biotechnologies	Cat# sc-17764
Tubulin β 3 (TUBB3) (TUJ1)	BioLegend	Cat# 801202
VECAD	R&D Systems	Cat# AF938
β-Actin	Invitrogen	Cat# am4302
**Critical commercial assays**		
Criterion 4-15% TGX precast gel	BioRad	Cat# 5671084
LDH-Glo Cytotoxicity Assay	Promega	Cat# J2381
Pierce BCA Protein Assay	Thermo Scientific	Cat# 23223
**Experimental models: Cell lines**		
A53T-1A CORR28	Harvard Medical School	RRID: CVCL_E3CE
AG09173 iPSCs	Massachusetts Institute of Technology	RRID: CVCL_4L66
ADRC5 iPSCs	UCI	
KOLF2.1J iPSCs	Jackson Laboratories	RRID:CVCL_B5P3
Recombinant DNA		
piggyBac-rtTA (4th_Gen)-NGN2-2A-PURO-IRES-SNAP	Addgene	Cat# 209077
piggyBac-rtTA (4th_Gen)-SNCA(A53T)-sfGFP-RES-NGN2-puro	Addgene	Cat# 209080
piggyBac-rtTA (4th_Gen)-SNCA(A53T-DNAC)-sfGFP-IRES-NGN2-puro	Addgene	Cat# 209081
PB_iETV2_P2A_GFP_Puro	Addgene	Cat# 168805
**Software and algorithms**		
Fiji	Schindelin, J. et al., 2012	imagej.net/software/fiji/
Nikon NIS Elements software	Nikon	microscope.healthcare.nikon.com
GraphPad Prism	GraphPad Software	graphpad.com
**Other**		
48-well plate	P48G-1.5-6-F	MatTek
96-well plate with cover glass thickness polystyrene bottom	Greiner Bio-One	655096
Ti2E Ax R Confocal Microscope	Nikon	microscope.healthcare.nikon.com
